# Deciphering RNA Regulatory Elements Involved in the Developmental and Environmental Gene Regulation of *Trypanosoma brucei*

**DOI:** 10.1371/journal.pone.0142342

**Published:** 2015-11-03

**Authors:** Vahid H. Gazestani, Reza Salavati

**Affiliations:** 1 Institute of Parasitology, McGill University, 21111 Lakeshore Road, Ste. Anne de Bellevue, Montreal, Quebec, Canada; 2 McGill Centre for Bioinformatics, McGill University, 3649 Promenade Sir William Osler, Montreal, Quebec, Canada; 3 Department of Biochemistry, McGill University, McIntyre Medical Building, 3655 Promenade Sir William Osler, Montreal, Quebec, Canada; Meharry Medical College, UNITED STATES

## Abstract

*Trypanosoma brucei* is a vector-borne parasite with intricate life cycle that can cause serious diseases in humans and animals. This pathogen relies on fine regulation of gene expression to respond and adapt to variable environments, with implications in transmission and infectivity. However, the involved regulatory elements and their mechanisms of actions are largely unknown. Here, benefiting from a new graph-based approach for finding functional regulatory elements in RNA (GRAFFER), we have predicted 88 new RNA regulatory elements that are potentially involved in the gene regulatory network of *T*. *brucei*. We show that many of these newly predicted elements are responsive to both transcriptomic and proteomic changes during the life cycle of the parasite. Moreover, we found that 11 of predicted elements strikingly resemble previously identified regulatory elements for the parasite. Additionally, comparison with previously predicted motifs on *T*. *brucei* suggested the superior performance of our approach based on the current limited knowledge of regulatory elements in *T*. *brucei*.

## Background

The unicellular flagellar parasite, *T*. *brucei*, causes sleeping sickness in humans and Nagana disease in cattle. Fine regulation of gene expression is a key challenge for *T*. *brucei* cells to adapt and survive in extremely variable environments and conditions as they shuffle from human host (bloodstream form) to tsetse fly vector (procyclic form) [1]. In trypanosomatids, unlike most eukaryotic cells, gene regulation almost exclusively occurs at the post-transcriptional level. RNA binding proteins (RBPs) have been found to mediate various crucial processes in these organisms, including developmental changes and cell cycle progression (for recent reviews see [2, 3]). During recent decades, much effort has been made to find and characterize the RBPs and their associated cis-acting RNA regulatory elements (RREs) in *T*. *brucei*. This has led to the characterization of RREs located in the 3′- untranslated regions (3′-UTRs) of different genes ([4–6] and references in [7, 8]), yet the gene regulatory map of the parasite remains mainly elusive.

Various computational approaches have been developed and applied for the genome-wide identification of RREs (reviewed in [9]). In particular, approaches based on whole genome expression profiling have proved powerful to infer these elements, leading to the identification of many established, as well as new, RREs [10–15]. Experimental results substantiate the view that many of the newly identified regulatory elements by these approaches are functional and can be recognized by the proteins on the genome [16].

Some expression-based computational approaches make predictions based on a single transcriptome experiment [10, 12, 13], while others decipher RREs by seeking enriched or informative motifs in sets of genes with common regulators [11, 14, 15]. To find co-regulated genes, the latter approaches group genes according to their expression patterns based on a comprehensive transcriptome dataset that covers a wide range of diverse biological conditions. Although powerful, the lack of comprehensive transcriptome data has greatly hampered their application on non-model organisms including trypanosomatid parasites. In the case of *T*. *brucei*, there is several transcriptome datasets each with a relatively small numbers of samples gathered from different experimental conditions.

To tackle the problem of RRE inference in *T*. *brucei*, we have developed a novel graph-based approach, termed GRAFFER, that identifies RREs by systematic integration of different transcriptome data sources. Application of GRAFFER to *T*. *brucei* transcriptome data led to the discovery of 88 RREs, of which eleven motifs resemble the previously known regulatory elements for the parasite. We also demonstrate that the novel elements not only agree with expected characteristics of functional RREs, but also are responsive to both transcriptomic and proteomic changes of the parasite during its life cycle.

## Materials and Methods

### Construction of the integrated co-expression graph

We focused on three independent transcriptome studies [17–19] to construct an integrated co-expression graph of *T*. *brucei*. To select for 25% genes with most variable expression patterns, we observed the variation of each gene (i.e., its standard deviation) in each dataset independently and the top 30%, 32% and 37% variable genes from [19], [17] and [18] were selected, respectively. The common protein coding genes among all three, consisting of about 25% of *T*. *brucei* genes (~1900 gene) were chosen for further analysis.

Initially, each of three microarray datasets were modeled as a weighted co-expression graph such that vertices represented genes, while their edges denoted the values of the pairwise Pearson correlation coefficient (PCC). The advantage of weighted co-expression graphs over un-weighted graphs is that the former preserve the underlying connectivity information. However, because the number of samples/conditions in each dataset is relatively small, weak correlations may not have biological relevance. To emphasize on strong correlations, we only considered those interactions which their squared values of the correlation coefficient (*r* ^2^) were equal or greater than 0.5. Moreover, negatively correlated pairs were excluded from each co-expression network as they do not support co-regulation. Next, an integrated co-expression graph was constructed by considering edges that are common in all three initial co-expression graphs. The edge weights of the integrated graph were defined as the average of weights for the corresponding edges in the three initial co-expression graphs.

Recently duplicated genes tend to have similar coding and 3′-UTR sequences. Thus, we would expect similar expression patterns for these genes in the microarray experiments because of cross-hybridization effects. Moreover, highly similar 3′-UTR sequences can cause bias in our motif scoring approach. To obviate these issues, from each two homologous genes that were present in the integrated co-expression graph, we randomly kept one and deleted the other one. Homologous genes in *T*. *brucei* genome were extracted from the MCL database v5.

### 3′-UTRs

3′-UTRs were defined according to the experimentally reported lengths by Siegel et al. [20]. However, in cases that gene had 3′-UTR of longer than 1000nt, the first 1000nt was considered for the prediction of RREs. The rationale behind the preference of such trimmed regions, instead of actual region of 3′-UTR is detailed in the S1 Text.

### Graph-based approach for finding functional elements in RNA (GRAFFER)

In current implementation of the GRAFFER algorithm, we considered only linear motifs generated over an alphabet of 11 characters (A, C, G, U, S = [CG], W = [AU], Y = [CU], R = [AG], M = [AC], K = [GU], N = [ACGU]). The terms “a gene harbors a motif” or “a gene targeted by a motif” were used, if the motif instance can be found in the 3′-UTR sequence of the gene. Accordingly, the module targeted by a motif is defined as the set of genes in the co-expression graph which are targeted by the motif.

Inspired by the cohesiveness concept in graph structure analysis [21, 22], GRAFFER quantifies the extent of connections between genes harboring the same motif by defining a motif modulation score, which is shown schematically in S1 Fig in S1 Text. The modulation score of a motif is defined as:

$$m=\frac{\sum_{intra-interactions}interaction\;weight}{\sum_{intra-interactions}interaction\;weight+\sum_{inter-interactions}interaction\;weight}$$

Where m represents the modulation score of the motif, intra-interactions are defined as the interactions of genes targeted by the same motif in the co-expression graph, and inter-interactions are defined as interactions of targeted genes with other genes (not targeted) in the graph.

To assess the statistical significance of observed motif modulation score for a motif, the corresponding Z-score was defined as:

$$Z-score=\frac{m\;-\;m_{0}}{sd}$$

Where m denotes the observed modulation score for the motif, and m_0_ and sd represent the expected modulation score and standard deviation for the motif with that redundancy, respectively (motif redundancy in a graph is defined as the number of the genes in the graph that are targeted by the motif). The expected modulation score and standard deviation for a motif of particular redundancy were estimated by observing the distribution of modulation scores for 1000 randomly selected modules of the same redundancy as the motif in the graph. For a given motif, GRAFFER estimates the Z-scores by assuming a normal distribution for the modulation scores. The distributions of the modulation scores are dependent on the graph structure; however, our preliminary results based on Kolmogorov-Smirnov goodness-of-fit test showed that only extreme cases, i.e. motifs with very high or low redundancy in the graph, violate normal approximation. Therefore, a minimum and maximum acceptable number of occurrences for each motif are considered for the analysis. Each acceptable motif should target at least 20 and at max (*n* − 20) genes in a co-expression graph with *n* nodes (e.g., genes). The lower limit will not cause a problem in our motif searching procedure because our goal is finding genome-wide conserved RREs.

A large scale experiment on a very diverse set of RBPs has demonstrated that these proteins tend to recognize and bind to short motifs with optimum predictive power at length of seven [23]. The short length of binding site is also supported with crystallography data [24, 25]. The same binding characteristic is also supported for trypanosomatid RBPs [7]. To search for motifs that target significantly dense modules in the co-expression network, GRAFFER starts by considering all possible 7-mer consensus patterns generated over an alphabet of 11 characters (as described above), with at least 4 non-degenerate bases and acceptable redundancy in the graph. The modulation score and the corresponding Z-score for each acceptable motif are then calculated. GRAFFER, next, selects motifs with significant modulation scores (Bonferroni corrected p-value <0.01) for the optimization process. The optimization process allows expansion in the motif consensus lengths. GRAFFER optimized each significant motif *m* by considering all possible, up to 9-mer, consensus patterns (constructed over the same alphabet) with the conserved consensus of *m* and chose the most significant one (the one with the highest Z-score) as the optimized motif.

Finally, multiple optimized motifs can represent various derivative forms of a single RRE originated from different primary 7-mers. To avoid redundancy in the predicted motifs list, GRAFFER sorts optimized motifs based on their observed Z-scores. Starting from the most significant motif, it creates adjusted 3′-UTRs by masking all instances of the motif and then recalculates the Z-scores for the remaining motifs. Next, motifs are again sorted based on recalculated Z-scores and motifs that have lost their significant state after the masking procedure are discarded. This procedure is repeated for the next most significant motif. GRAFFER ends this cycle when no more significant motifs remained. As final report, GRAFFER reports back the motifs that remained significant in the above mentioned procedure. The employed procedure guarantees that each motif targets a significantly dense module in the co-expression graph and the significance state of each motif is independent of presence of the others.

### Motif co-occurrence profile

To identify combinatorial interactions among predicted motifs, we compared the probability of co-occurrence of two motifs to the expected probability of co-occurrence by chance. To estimate the expected probability of co-occurrences for two motifs **m**_**1**_ and **m**_**2**_, random motif pools for each **m**_**1**_ and **m**_**2**_ were considered, each composed of 200 random motifs with the same length and redundancy (i.e., the number of the genes in the graph that are targeted by the motif) as **m**_**1**_ and **m**_**2**_, respectively. We observed the expected probability of co-occurrence for **m**_**1**_ and **m**_**2**_ by examining each possible combination of corresponding random motifs present in their pools. By assuming that the null model follows a binomial distribution, we reported the Z-score for the pair of **m**_**1**_ and **m**_**2**_ as

$$\left(K-NP_{0}\right)/\sqrt{NP_{0}\left(1-P_{0}\right)}$$

where **K** denotes the common targets between **m**_**1**_ and **m**_**2**_; **N** represents total number of unique targets for **m**_**1**_ and **m**_**2**_; and **P**_**0**_ represents the expected probability of co-occurrence for motifs **m**_**1**_ and **m**_**2**_.

### Motif gene set enrichment analysis

To identify enrichment of a motif in a specific cell state in the proteome or transcriptome datasets, genes were ranked according to their normalized expression values. We then examined if the genes targeted by the motif showed statistically significant over-representation toward the top or bottom of the ranked list, using the standard Mann-Whitney rank sum statistic. The Benjamini-Hochberg false discovery rate of 0.05 was selected as the cut-off threshold.

## Results and Discussion

### Prediction of functional gene regulatory elements

To infer RREs involved in the developmental and/or environmental responses of *T*. *brucei*, we considered three independent genome-wide transcriptome studies on *T*. *brucei* that included different life stages [19], developmental processes triggered by the addition of cis-aconitate and lowering the temperature [17], and responses to a variety of chemical perturbations [18]. It is suggested that around 5–25% of trypanosome genes are responsive to the environmental changes. Hence, in our analysis, we focused on 25% most variable genes in terms of expression patterns as determined by the transcriptome data. As elaborated in the method section, given the three microarray datasets [17–19], we first modeled each dataset as a co-expression graph, where vertices represented genes and edges represented co-expression over the dataset. Next, an integrated co-expression graph was constructed by considering edges that were present in all three initial co-expression graphs. Switching from expression profiles to co-expression graphs proved to be an efficient way to identify sets of co-expressed genes across multiple datasets [26]. Preliminary topological analysis of the integrated co-expression graph revealed that it exhibits both striking characteristics of most biological networks, small-world behavior and the scale-free property (S2 Fig in S1 Text).

**Fig 1:**
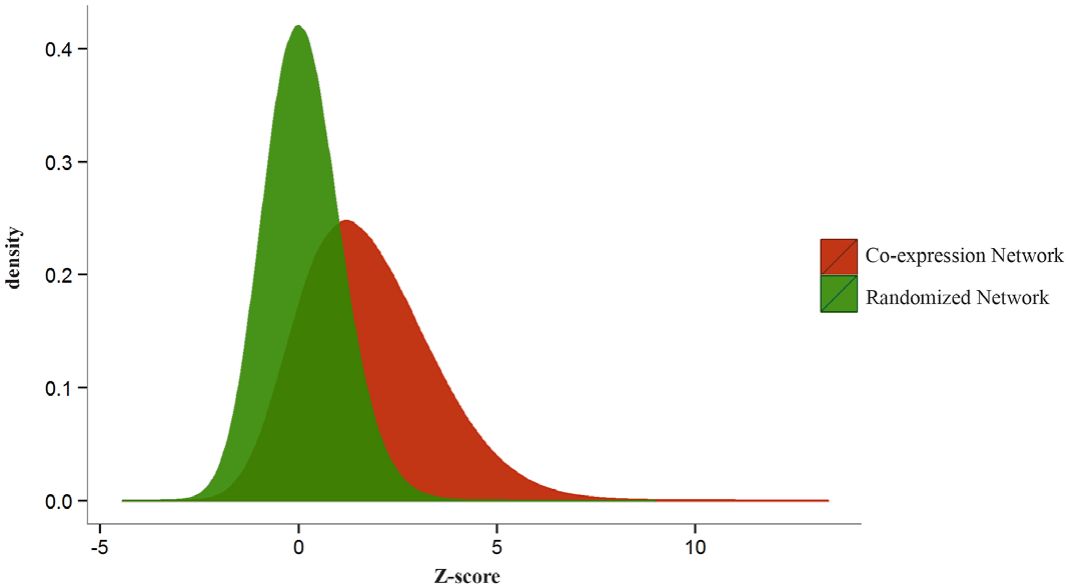
Distribution of motif modulation scores for the integrated co-expression graph and a random graph. As illustrated, the distribution of motif scores for the integrated co-expression graph is right-skewed compared to that of the random graph.

Recent studies demonstrated that most RBPs recognize single stranded, linear RNA sequences and the structure around a binding site is mainly to support its single strandedness [23, 27]. Studies on RREs in *T*. *brucei* have led to a similar idea; although there can be some structures associated with the functional regulatory sites [4, 5, 28], these structures may not be conserved for the corresponding RREs [4]. Therefore, in current work, we focused on linear sequence motifs for the identification of potentially functional RREs. Moreover, RREs tend to be enriched in the 3′-UTR region of trypanosomatid genes, although exceptions for some RREs (like prominent presence in the coding sequence) have been reported [29]. Here on, the terms “a gene harbors a motif” or “a gene targeted by a motif” were used, if the motif instance can be found in the 3′-UTR sequence of the gene (The employed approach for the selection of 3′-UTR regions are detailed in S1 Text). To discover linear RREs that target a set of coherently expressed genes, we developed a novel method, called GRAFFER, to search for linear motifs whose targeted genes create a significantly dense module in the co-expression graph. To predict functional RREs, GRAFFER calculated the module density of more than 4 ×10^6^ distinct linear motifs in the case of *T*. *brucei* integrated co-expression network. To assess the discriminative power of the defined score on the integrated co-expression graph of *T*. *brucei*, we compared the distribution of scores in this graph with a random graph, constructed by random permutation of gene labels in the integrated co-expression graph. As shown in Fig 1, the distribution of scores for motifs in the co-expression graph is right-skewed, while the distribution of scores for the same set of motifs in the random graph is randomly distributed. This figure clearly shows that the co-expression graph conveys information (based on the defined score) that is absent in the random graph. Application of GRAFFER to *T*. *brucei* integrated co-expression graph led to the prediction of 88 non-redundant motifs whose targeted genes were significantly connected to each other in the graph (Bonferroni corrected p-value <0.01; S1 Table and S3a Fig in S1 Text). However, applying GRAFFER with the same settings to 100 random networks, generated by random shuffling of gene labels in the co-expression graph, yielded 9.6 motifs on average (The employed randomization procedure changes the location of 3′-UTRs in the graph, while preserve the graph topological characteristics). The connection of a pair of genes in the integrated co-expression graph indicates their co-expression under various conditions; therefore, the significance of predicted motifs implies that the corresponding targeted genes by these motifs tend to be significantly co-expressed with each other under a wide range of conditions. As is expected from RREs, directional analysis of GRAFFER motifs showed that they mostly (more than 95%) have a strand bias and are significant only in the forward strand (S3b Fig in S1 Text).

To systematically estimate the accuracy of GRAFFER predictions, we applied the approach on human, for which many RREs are experimentally identified, providing a rich context to systematically examine the accuracy rate of the approach. As elaborated in the S1 Text, application of the GRAFFER to human led to the prediction of 49 significant motifs (S2 Table). The GO enrichment analysis of these motifs revealed that 37 out of 49 predicted motifs target transcripts significantly enriched for at least one biological process (S4 Fig in S1 Text). Furthermore, we found that 27 motifs resemble experimentally identified regulatory elements recognized by the RBPs and/or miRNAs, many of which are significantly enriched in the 3′-UTR of transcripts that are targeted by the RBPs and/or miRNAs (S5 and S6 Figs in S1 Text). These results demonstrated that 55% of motifs that were predicted by GRAFFER, matched with the previously known, experimentally-derived RREs, proving underestimated accuracy for our approach (Some of unverified predictions of GRAFFER for human might be correct, but not experimentally identified yet).

### Characteristics of the predicted RREs

An experimentally deciphered regulatory network of 40 different RBPs in *Saccharomyces cerevisiae* revealed a complex combinatorial network among RBPs, such that different RBPs can target a similar set of RNAs [30]. The existence of such extensive regulatory networks is also suggested in *T*. *brucei* [28, 31]. To explore the putative relationships among the predicted motifs, we examined the existence of significant patterns of co-occurrence for each pair of motifs (detailed in the method section). As shown in S7 Fig in S1 Text, 29 pairs showed significant co-occurrence patterns with each other. This result supported the hypothesis that gene expression in *T*. *brucei* is regulated by a complex regulatory network. Lack of co-occurrence patterns for other motifs also indicated they target distinct sets of genes, suggesting diverse biological roles for them.

**Fig 2:**
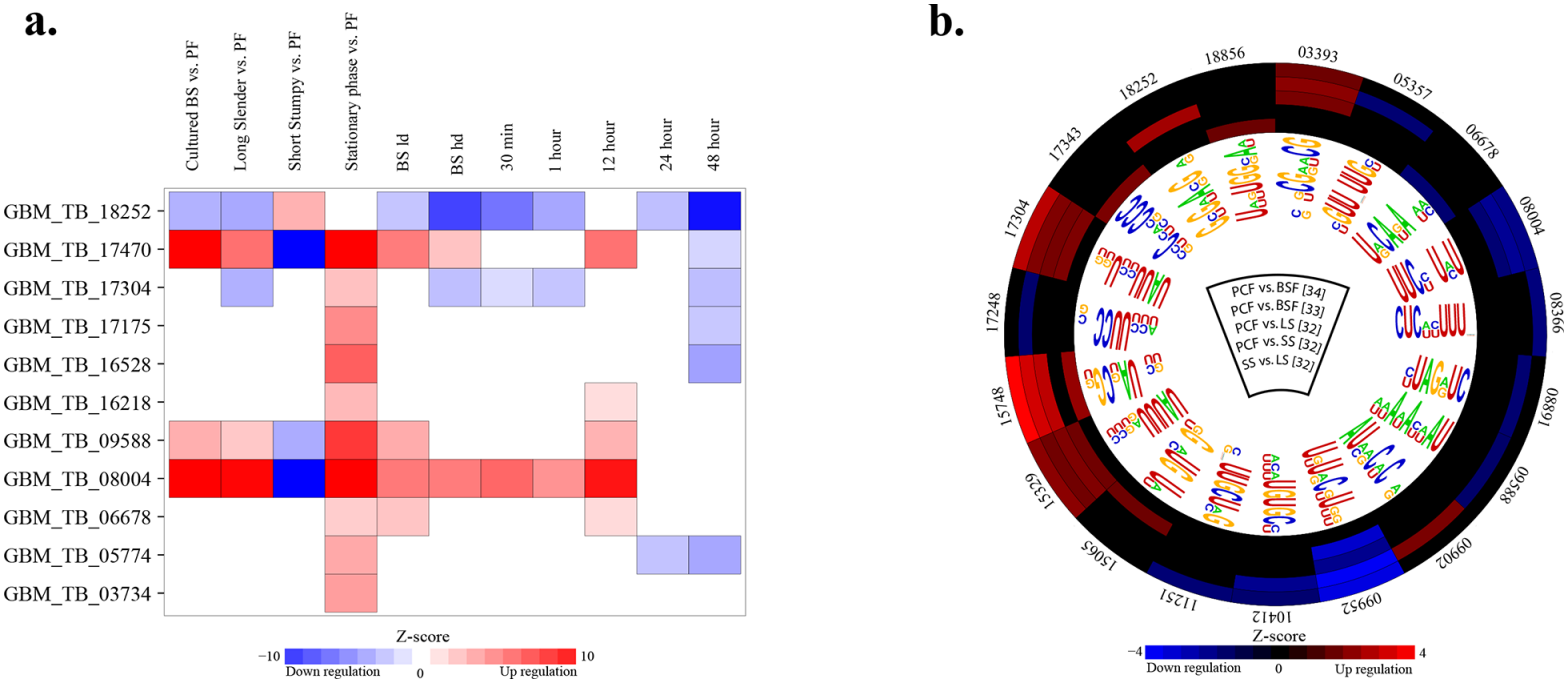
Motif enrichment analysis using available transcriptome and proteome data of *T*. *brucei*. For each GRAFFER motif, transcripts containing the motif in their 3′-UTR were selected and then tested for a statistically significant pattern in each cell state using standard Mann-Whitney rank sum statistic. (a) Developmental transcriptome responses of the eleven GRAFFER motifs that matched with the previously known RREs in *T*. *brucei*. For illustrative purposes, the full transcriptome enrichment analysis for the rest of the predicted motifs is not represented here. The complete results of this analysis are illustrated in S8 and S9 Figs. (b) Proteome enrichment analysis revealed that 19 motifs showed significant enrichment under at least one condition. The outer layer of circle indicates the motif id, excluding “GBM_TB” term due to illustration limitations. The intermediate circular layers indicate the up- or down-regulation of the motifs in a specific condition. The inner circular layer represents the consensus patterns of motifs. The center of circle shows the conditions that were tested for the enrichment with the reference for the study that data was extracted from.

Additionally, we examined whether the predicted motifs showed specific expression patterns in the transcriptome data of each cell state. This analysis revealed that 84 out of 88 (95%) predicted motifs showed significant enrichment under at least one condition (Fig 2a and S8 and S9 Figs in S1 Text). We also considered the available proteomics data [32–34] to further demonstrate the functionality of predicted motifs at the proteome level. Following the same approach as the transcriptome data, we found that 19 motifs (22%) showed significant enrichment under at least one condition (Fig 2b). Notably, the available proteomics data, compared with the transcriptome data, were from a limited set of conditions. Therefore, we can expect this number to increase as more proteomic data becomes available. As discussed later, we show that enrichment results for several motifs are consistent with previous knowledge on the gene regulatory network of *T*. *brucei*.

### Comparison of predicted RREs with previously identified or predicted regulatory elements

**Fig 3:**
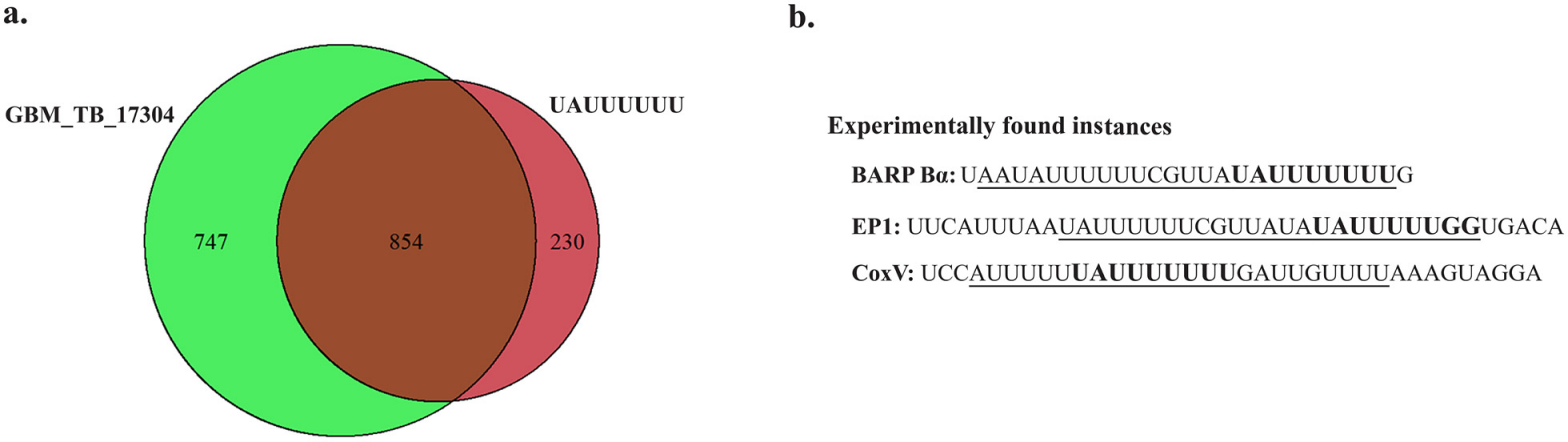
Developmentally regulated U-rich RRE in *T*. *brucei*. Comparison of an experimentally established RRE (UAUUUUUU) that is involved in developmental regulation of *T*. *brucei* genes, with GRAFFER motif, GBM_TB_17304. (a) Venn-diagram of the transcripts that are targeted by UAUUUUUU and GBM_TB_17304 motifs. (b) Underlined regions show the U-rich regions (extracted from [35, 36]) with the experimentally-verified regulatory role that were used to infer UAUUUUUU regulatory element. The bold sequence in each U-rich region represents the part region that matches with GBM_TB_17304 motif.

To date, only a small fraction of RREs in *T*. *brucei* have been identified. Hence, we could not compare GRAFFER results with a large number of experimentally-derived regulatory elements. However, the fifth most significant motif (GBM_TB_17304) shows close similarity with one of the most intensely studied U-rich RREs in *T*. *brucei* [35, 36]. The predicted motif not only strikingly resembles the experimentally derived RRE, but also has highly overlapping RNA targets with it (Fig 3a). It is suggested that the experimentally determined RRE targets the 3′-UTR of many diverse sets of transcripts on a genome-wide scale [35, 36]. From the functional perspective, it is experimentally verified that this RRE is involved in the developmental regulation of transcripts, with a destabilizing effect on target RNAs in the bloodstream form (S10 Fig in S1 Text). Intriguingly, the GBM_TB_17304 motif also showed a similar effect on the targeted RNAs at both transcriptome and proteome level (S10 Fig in S1 Text). More importantly, GBM_TB_17304 matches with the previously known functional instances of the experimentally derived regulatory elements (Fig 3b). These lines of evidence indicate that potential follow up experimental works on GBM_TB_17304 can lead to the same biological knowledge as that of the experimentally derived RRE.

Additionally, another predicted motif (GBM_TB_16528) resembles a previously identified A/U-rich element that is involved in the heat-shock response of the parasite [6]. It is experimentally verified that *ZC3H11* zinc finger protein can recognize and bind to the A/U-rich element with stabilizing effect on the target RNAs during heat stress. Congruent with the experimental evidence, genes targeted by GBM_TB_16528 are gradually down-regulated during the differentiation of bloodstream cells to procyclic cells where along with the change of cell media, the temperature is reduced from 37°C to 27°C with most reduction in expression level observed after 48hrs of differentiation process (p-value <4E-05; Mann-Whitney rank sum test). Moreover, Genes targeted by GBM_TB_16528 also show moderately reduced expression patterns in cultured bloodstream forms compared to the cultured procyclic cells (p-value <0.085; Mann-Whitney rank sum test). The bound transcripts to the *ZC3H11* have been also reported using cross linking experiments coupled with deep sequencing [6]. Importantly, GBM_TB_16528 is significantly enriched among the strongly bound transcripts to the *ZC3H11* (p-value < 2E-13; two tailed hypergeometric test) and matches with the known instances of experimentally-determined A/U-rich element, as reported in [6].

Benefiting from an *in vitro*, Selex-based technique known as RNAcompete [37], a large-scale study has revealed the binding preference of 13 trypanosomatid RBPs [23]. As detailed in S1 Text, comparison of the motifs revealed that 11 out of the 13 trypanosomatid Selex-based motifs showed significant similarity to nine out of 88 GRAFFER motifs (S3 Table). Matching the RNAcompete results with the GRAFFER predictions also led to new insights on the functional roles of the RBPs. As an illustration, the comparison suggested the recognition of GBM_TB_09588 motif by *DRBD13* protein (*Tb927*.*8*.*6650*). A recent study has experimentally demonstrated that *DRBD13* protein is essential for the procyclic life stage of the parasite and its tethering to RNA leads to the down regulation of the target in this life stage [38]. Consistent with this, we found transcripts harboring GBM_TB_09588 are significantly down regulated in the procyclic stage compared to both long slender stage (p-value < 0.002; Mann-Whitney rank sum test) and cultured booldstream cells (p-value <0.0002; Mann-Whitney rank sum test). Additionally, re-analysis of available *DRBD13* tandem affinity purification coupled with deep sequencing (RIP-seq) data [18] indicated that GBM_TB_09588 is significantly enriched among the bound transcripts to the RBP (p-value <4E-32; Mann-Whitney rank sum test). Moreover, congruent with suggested role of *DRBD13* protein on regulation of membrane associated proteins [38], genes targeted by GBM_TB_09588 are significantly enriched for genes involved in antigenic variation (p-value <8.30E-16; Fisher exact test). As another example, the comparison of RNAcompete results with GRAFFER predictions demonstrated the possible recognition of GBM_TB_16218 motif by *DRBD12* protein (*Tb927*.*7*.*5380*). Two previously published studies have experimentally demonstrated the destabilization role of *DRBD12* protein on its target [18, 39]. Consistently, re-analsis of available microarray data on knock down of *DRBD12* indicated that genes targeted by GBM_TB_16218 are significantly up-regulated in its knock down background (p-value < 0.0007; Mann-Whitney rank sum test). Moreover, re-analysis of available RNA-seq data on the insect-stage life-cycle of *T*. *brucei* demonstrated about three fold up-regulation of *DRBD12* in the proventriculus life stage [40]. Consistent with destabilization role of *DRBD12* protein, transcripts containing GBM_TB_16218 motif are significantly down regulated in this life stage (p-value <0.02; Mann-Whitney rank sum test). This data suggests the possible role of *DRBD12* RBP in the insect stage differentiation process of the *T*. *brucei*.

It worth noting that all four motifs discussed above have more than fifty percentages of A and/or U in their consensus sequence. However, as discussed above, they show diverse responses during the life cycle of the parasite. GBM_TB_17304 motif is significantly upregulated in the procyclic cells. In contrast, the two motifs of GBM_TB_09588 and GBM_TB_16528 are upregulated in the bloodstream life stage, while their mechanisms of actions are different than each other which is also supported by motif co-occurrence profiles. In fact, 64% (56 out of 88) of predicted motifs by GRAFFER have more than 50% A and/or T in their composition. However, these motifs mostly target different transcripts (as judged by motif co-occurrence profiles) and show different responses during the life cycle of *T*. *brucei*, suggesting a potentially distinct and diverse role for A- and/or U-rich motifs in the gene regulatory network of the parasite.

Based on the available experimentally verified RREs, we compared the performance of GRAFFER with three other genome-wide computational studies of RREs on *T*. *brucei* [18, 41, 42]. The comparison showed that our new approach outperformed all of them in terms of accuracy. Briefly, no experimental motifs with a developmental role were predicted by any of them. In addition, the comparison of RNAcompete-derived RREs with the predicted motifs from each article revealed that the RNAcompete RREs had better agreement with our new approach compared with the others (see S1 Text and S4 Table for the details). It is important to note that RREs are not extensively characterized in *T*. *brucei*; which led us to compare the predictions of each study with a limited set of previously known RREs. Therefore, some of the novel RREs predicted by these approaches may be valid, but not discovered yet.

### Assessment of the dataset integration performance

**Fig 4:**
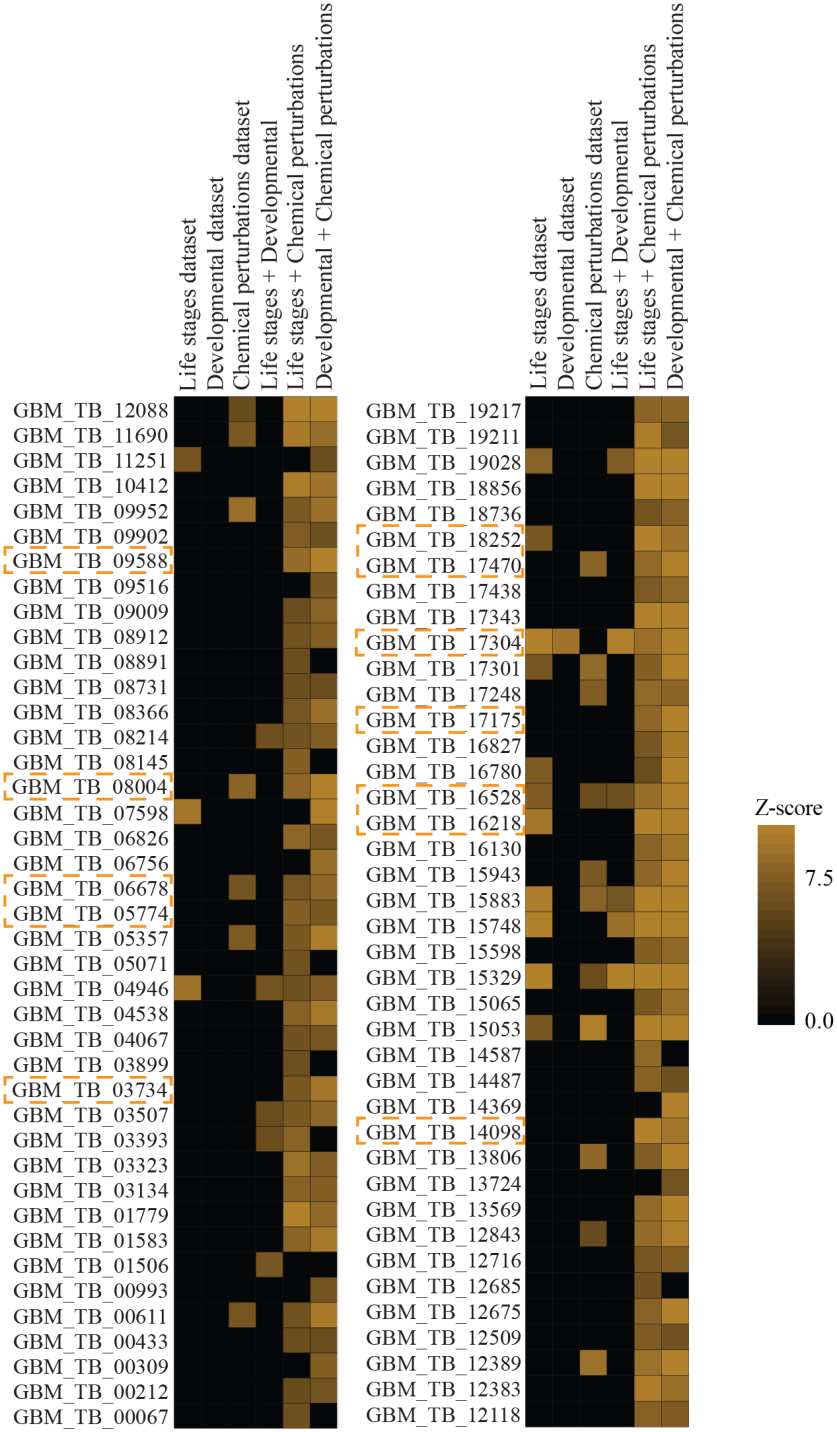
Performance of GRAFFER using only a subset of datasets. The columns represent six different co-expression graphs that were constructed by considering one or two transcriptome datasets. The figure is pseudo-colored, with only conditions (i.e., co-expression graphs) that motif has significant Z-scores are only shown. The orange boxes around some of the motif names highlight the motifs that matched to the experimentally validated RREs.

To further evaluate the performance of our approach, we investigated the significance of predicted motifs in cases where we constructed a co-expression graph by considering only a subset of datasets (one or two datasets) instead of all three. It should be pointed that application of GRAFFER in these cases may lead to the prediction of some new motifs; however, because the number of samples in each dataset is relatively small, it is likely that regulatory circuits are not well separated from each other, leading to the connection of genes involved in parallel regulatory circuits in these graphs. Therefore, we only focused on the set of 88 motifs, ignoring the newly found instances in each case as they are not most likely reliable. As shown in Fig 4, each dataset on its own was not informative enough and few motifs remained significant in these networks (1% developmental, 16% life stages, and 20% chemical perturbations). However, most motifs became significant when the co-expression graph was constructed by integrating at least two datasets. This result was anticipated, because each dataset on its own had captured only a small set of cell states, but when the datasets were integrated with each other, the responses of the cell system became more evident, leading to the prediction of more significant motifs. Interestingly, we found that the integration of two contextually dependent datasets (developmental and life stages datasets) did not improve the performance of approach noticeably; however, the integration of two contextually independent datasets (life stages and chemical perturbations datasets or, alternatively, developmental and chemical perturbations datasets) boosted the inference power of the approach significantly. Previous studies suggested that although there is a significant gene expression remodeling in different life stages and during differentiation process, gene expression variations within each life stage are limited. This assumption has meant that almost all genome-wide studies (transcriptome and proteome) have focused on the developmental aspects of gene expression. In our analysis, we clearly observed that the integration of development-related datasets with a limited dataset (11 samples) from chemical perturbations increased the precision of co-expression graph dramatically. This analysis also provided insights into the functional regulatory roles of some of the predicted RREs. For example, developmentally regulated RRE (GBM_TB_17304) is significant in both the developmental and life stages datasets; however, it loses its significance in the chemical perturbations dataset.

Independent application of GRAFFER on each of the three datasets [17–19] indicated its power to find functional RREs from datasets with a relatively small number of samples, but limited relative to the case that datasets were integrated with each other. To further test this hypothesis, we considered the available cell cycle gene expression data in *T*. *brucei* [43], comprised of four cell states (Early G1, Late G1, S phase, and G2/M phase). Consistently, as elaborated in the supplementary information (S1 Text; S5 Table), we found that GRAFFER predictions in this case show close similarity with experimentally validated RREs involved in cell cycle regulation in trypanosomatid organisms, supporting the potential predictive power of our approach on limited datasets.

## Concluding remarks

In this study, we have introduced a graph-based solution to predict RREs by systematic integration of different transcriptome data sources. This property becomes particularly important in the study of non-model organisms with limited whole genome expression datasets. Application of our approach has led to the prediction of 88 RREs that function in the gene regulatory network of *T*. *brucei*. To date only a small fraction of RREs in *T*. *brucei* have been identified, yet eleven predicted motifs strikingly resemble experimentally-derived trypanosomatid regulatory elements. Further comparison of these eleven motifs with experimentally-derived RREs indicated that they not only target highly overlapping transcripts, but also show similar transcriptome and proteome responses to the environmental and developmental changes of *T*. *brucei*.

Application of GRAFFER on random graphs suggested false discovery rate of less than eleven percent for the predictions, suggesting a high accuracy rate for the predictions. Additionally, application of GRAFFER to human demonstrated that 55% of predictions match to previously known RREs. Moreover, our results indicated that 95% of the predicted motifs for *T*. *brucei* are responsive to the transcriptome and proteome changes in the life cycle of the parasite. In several cases, we have shown that these predictions match with previous knowledge on the gene regulatory network of *T*. *brucei*. Our results also led to the prediction of biological roles for several uncharacterized RREs and RBPs.

Consistent with experimental evidences [31], the motif co-occurrence patterns suggested intricate and intertwined regulatory relationship between some of the regulatory elements and, consequently, their cognate RBPs. However, these patterns also revealed that many motifs target distinct RNAs, suggesting regulation of a wide range of different trypanosomatid RNAs by RBPs. Moreover, the sequence characteristics of the predicted motifs highlight the importance of A- and/or U-rich elements in the gene regulatory network of *T*. *brucei*. Importantly, these motifs show distinct transcriptome and proteome responses to the life cycle changes of the parasite, suggesting their diverse regulatory roles.

Although GRAFFER is designed to allow inference of RNA regulatory elements based on limited transcriptome data sources by their systematic integration, it still relies on the concept of co-expression. The approach first models each dataset as a graph where edges represent co-expression over the dataset. The initially constructed co-expression graphs are then systematically integrated to gain a higher resolution picture of underlying regulatory circuits in the cells. Our analysis clearly demonstrated that while inference of regulatory elements based on a single dataset provided limited information, the integration step boosted the inference power significantly. However, due to reliance on the co-expression concept for the construction of the initial co-expression graphs, datasets with extremely limited number of sample (i.e. those monitoring expression changes in only two or three different conditions) cannot be used in our approach. Therefore, to infer RREs that are responsive to the developmental and/or environmental changes of the parasite, we have only considered transcriptome datasets that capture gene expressions in at least five biologically different conditions.

Experimental knowledge on mechanisms of actions of RBPs demonstrates that the recognized elements by these proteins are either single stranded or have particular secondary structures. Therefore, the secondary structure of RNAs play important role in the recognition of RREs by the RBPs. However, the current implementation of GRAFFER focuses only on the 3’-UTR sequences, ignoring the RNA secondary structures. Therefore, the 88 predicted are biased towards the linear motifs with no knowledge on the structural context of the motif instances present in the 3’-UTRs. Although recent studies demonstrated that most RBPs recognize single stranded RNA sequences and the structure around a binding site is mainly to support the single strandedness [23, 27], the employed motif searching procedure leads to systematic loss of structural dependent motifs, some of which are shown to play important roles in the gene regulatory of the parasite [4, 5, 28].

Algorithm availability. We have implemented a C# package for the GRAFFER algorithm, available at http://TrypsNetDB.org/graffer.zip. The input data is straightforward. It only needs a weighted co-expression graph and the UTR sequences.

## Supporting Information

S1 Table GRAFFER motifs predicted based on the integrated co-expression graph of *T*. *brucei*. List of 88 significant GRAFFER motifs for *T*. *brucei* which were inferred based on the integrated co-expression graph.
(XLSX)

S2 Table GRAFFER motifs predicted based on a co-expression graph of human. List of 49 significant GRAFFER motifs predicted for Human.
(XLSX)

S3 Table Comparison of RNAcompete motifs with GRAFFER motifs. The detailed comparison of RNAcompete motifs with GRAFFER motifs which were predicted based on the integrated co-expression graph of *T*. *brucei*.
(XLSX)

S4 Table Comparative assessment of the motifs predicted in the three previous genome-wide analysis of *T*. *brucei* UTRs. Comparison of RNAcompete motifs with computationally predicted motifs from three previous studies.
(XLSX)

S5 Table GRAFFER motifs inferred based on the cell cycle co-expression graph of *T*. *brucei*. List of five significant motifs for *T*. *brucei* which were inferred based on the cell cycle co-expression graph of *T*. *brucei*. In this co-expression graph, genes with coherent transcriptome responses during cell cycle progression are connected to each other.
(XLSX)

S1 Text Supplementary methods and figures. This file contains supplementary methods and figures.
(PDF)
