# Supplementary material for: Deciphering RNA Regulatory Elements Involved in the Developmental and Environmental Gene Regulation of Trypanosoma brucei
Source: PLoS One. 2015 Nov 3;10(11):e0142342. doi: 10.1371/journal.pone.0142342 (PMC4631447; doi:10.1371/journal.pone.0142342)
Supplement: S1 Text — This file contains supplementary methods and figures. (PDF) [file pone.0142342.s006.pdf]

Supplementary Information

Deciphering RNA regulatory elements involved in the gene regulation of *Trypanosoma brucei*

Vahid H. Gazestani[1], Reza Salavati[1,2,3,*]

[1]Institute of Parasitology, McGill University, 21111 Lakeshore Road, Ste. Anne de Bellevue, Montreal, Quebec H9X3V9, Canada

[2]McGill Centre for Bioinformatics, McGill University, 3649 Promenade Sir William Osler, Montreal, Quebec H3G0B1, Canada

[3]Department of Biochemistry, McGill University, McIntyre Medical Building, 3655 Promenade Sir William Osler, Montreal, Quebec H3G1Y6, Canada

[*] Corresponding author

Benchmarking GRAFFER on human

To search for RREs in human, we constructed a co-expression graph based on a compendium of 211 expression profiles across 38 distinct human hematopoietic cells, monitoring gene expression changes during the hematopoietic differentiation process (1). The interaction density of human co-expression graph was similar to the case of *T. brucei* integrated co-expression graph; and weights of edges were defined by Pearson correlation coefficient. 3′-UTRs of human genes was defined the immediate 300nt down-stream of stop codon in the longest isoform of transcript, as described elsewhere (2). The terms “a gene harbors a motif” or “a gene targeted by a motif” were used, if the motif instance can be found in the 3′-UTR sequence of the gene. Accordingly, a module targeted by a motif is defined as the set of genes in the co-expression graph which are targeted by the motif.

Application of GRAFFER led to the prediction of 49 significant non-redundant motifs whose targeted genes were significantly connected to each other in the co-expression graph of human, with Bonferroni corrected p-value less than 0.01 (S11.a Fig and S2 Table). As expected for RREs, directionality analysis of GRAFFER motifs demonstrated that 47 motifs (~96%) show a strand bias and are significant only in the forward strand (S11.b Fig).

The predicted motifs target 49 densely connected modules in the co-expression graph. To assess the biological relevance of predicted modules, we first examined whether or not these modules were enriched for specific gene ontology (GO) biological process terms. This analysis revealed that 37 out of 49 predicted modules were enriched for at least one biological process (S4 Fig), suggesting that although the modules were predicted solely based on characteristics of the 3′-UTR sequences and the co-expression graph, they have specific functions in the cell.

The recent large scale RNAcompete study has identified the binding preference of 205 distinct RBPs (3). This study also predicted a high confidence regulatory network for some of human's RBPs based on the integration of information on RREs and available transcriptome dataset (3). As illustrated in S5 Fig and detailed in RNAcompete section of supplementary text, comparison of predicted motifs with those of RNAcompete showed that 24 GRAFFER motifs are significantly similar to 62 RNAcompete experiments (S2 Table; some of the RNAcomplete experiments had replicates or identified the binding preference of several orthologous RBPs, leading to the matching of some GRAFFER motifs with multiple RNAcompete-derived motifs). Consistently, in cases that a GRAFFER motif matched with the binding site of an RBP with available predicted target RNAs, the predicted motif was significantly enriched in the 3′-UTR of the predicted targets as well.

To test whether the GRAFFER motifs can be related to miRNAs, we first examined if there is enrichment for the predicted targets of human miRNAs in the 49 found modules. This analysis showed that 42 modules (~86%) are enriched for the target RNAs of at least one human miRNA. Congruent with evidences about the complex interplay between RBPs and miRNAs (4, 5), we found that many of modules that were predicted to be regulated by RBPs in the previous step can also be regulated with at least one miRNA. Moreover, we found that for 7 motifs, not only the cognate module is enriched for the target RNAs of a specific human miRNA, but also the motif match to the 5′- extremity of the miRNA (S6 Fig; It should be noted that only human miRNAs were considered for matching with GRAFFER motifs). Interestingly, four of GRAFFER motifs that matched with human miRNA binding sites, showed significant similarity to the RBP binding sites as well which can be suggestive of potential competition for binding between RBPs and miRNAs.

The obtained results from this analysis demonstrated the power of our graph-based approach in identification of functional RREs based on co-expression graphs.

Application of GRAFFER to Cell cycle transcriptome

For the *T. brucei* cell cycle co-expression graph, we extracted expression profiles from (6) and considered genes that showed at least a 1.5 fold change in one cell cycle stage compared with early G1 phase. This dataset comprised of four cell states, monitoring gene expression as *T. brucei* cells move through cell cycle (Early G1, Late G1, S phase, and G2/M phase). Performing the same steps as our previous attempt, we applied GRAFFER on the constructed co-expression graph from this dataset. In this case, our approach identified five significant motifs (S12.a Fig and S5 Table). The low number of significant motifs was anticipated because of the low number of samples in the dataset. Comparison of the predicted motifs with experimentally established motifs revealed that one of our motifs matched a well-studied RRE in trypanosomatids. This experimentally validated RRE is involved in cell cycle regulation in trypanosomatid organisms (7). Importantly, genes harboring each of these experimental and computational motifs were significantly upregulated in the late G1 cell cycle phase (S12.b Fig).

T. brucei 3′-UTR sequences

The 3′-UTR sequences were downloaded from TriTrypDB v.5, considering lengths reported in (8). In cases of alternative poly-adenylation, the median length was selected. In cases that gene did not have an identified 3′-UTR length, 400nt (the median 3′-UTR length of *T. brucei* genes) downstream of the translational stop codon was selected. Preliminary analysis of 3′-UTR lengths

revealed that although the median length is 400nt, some transcripts can have very long 3′-UTRs (S13.a Fig). Recent discoveries suggested that alternative poly-adenylation site selection can have regulatory impact on the expression level of transcripts in different organisms (9). For transcripts with alternative 3′-UTRs, the longer isoforms potentially have more binding sites for RNA-binding proteins and/or miRNAs. In general, the outcome of having more regulatory regions is that isoforms with shorter 3′-UTRs have elevated expression levels compare with the longer isoforms of the same transcript (10). In support to the regulatory role of alternative poly-adenylation site selection, the 3′-UTR length of at least one transcript in *T. brucei* is reported to be developmentally regulated (11). Moreover, alternative trans-splicing (which can lead to variation in 3′-UTR lengths) plays a role in the developmental regulation of some *T. brucei* genes (12).

Previous studies on *T. brucei* suggested that poly-adenylation site selection in this organism is linked to the selection of the downstream 3′-splice-acceptor site **(13)**. Considering both dependency on splice-acceptor-site selection and the error in sequencing that may occur because of the low complexity of 3′-UTR regions, the existence of minor variations on detected poly-adenylation sites was anticipated. To test the possibility that gene expression is regulated by alternative poly-adenylation site selection, we first examined the agreement between two published studies on poly-adenylation sites of *T. brucei* transcripts **(8, 14)**. Considering each study independently, we defined poly-adenylation regions by considering ±50nt around each detected poly-adenylation site. If two adjacent poly-adenylation sites had overlapping regions, relevant regions were merged and the new region was defined as the union of both. Thus, two poly-adenylation sites in different regions would be at least 100nt far from each other, shown schematically in S13.b Fig. By applying this selection criterion, we tolerated false negative

results to reduce false positives. This analysis revealed that for many genes in *T. brucei*, there are at least two poly-adenylation regions supported by two independent studies (S13.c Fig). Next, we examined the agreement of 3′-UTR length variation for transcripts with at least two poly-adenylation regions in both studies. Considering standard deviation of 3′-UTR length variation obtained from each article, we observed a moderate but significant agreement for 3′-UTR length variation between the two studies (S13.d Fig). This result demonstrated that 3′-UTR length variation is replicable and two independent experiments with different coverage levels produced similar results. Intriguingly, we found that although transcripts with very long 3′-UTRs (length > 1000nt) are usually downregulated under most biological conditions (as expected); these genes are significantly upregulated in some specific stress conditions (S14 Fig). Coherent upregulation of these genes under some stress conditions could occur by disruptions in 3′-UTR length regulation mechanisms under these stress conditions or by up- or downregulation of some specific RBPs that mediate 3′-UTR length variation in response to the stress. Considering 3′-UTR lengths according to **(8)**, statistical analysis of transcripts with long 3′-UTRs (length > 1000nt) showed that these transcripts have a significantly tendency to have more than one poly-adenylation region (Mann-Whitney rank sum, $\boldsymbol{p-value < 10^{-114}}$). Unfortunately, most poly-adenylation sites in *T. brucei* were detected in only one cell state (Procyclic form, log-phase). This restricted us to examining whether different isoforms of some transcripts are preferred in different cell states, but these data suggested that there may be other regulatory mechanisms in parallel to RREs, which regulate the expression levels of *T. brucei* genes, particularly for genes with long 3′-UTRs. Coherent up- or downregulation of these transcripts implies that they have predictable expression patterns, independent of their long 3′-UTR sequences. Besides, this coherency in expression patterns resulted in their significant connections

to each other in the constructed co-expression network ($\boldsymbol{p-value} < \mathbf{10^{-34}}$). The significant connections of these transcripts to each other along with their long 3′-UTRs could compensate for the random distribution of some non-functional motifs, leading to a bias in our motif prediction approach. To take these issues into account, we restricted the maximum 3′-UTR length for each transcript to 1000nt (i.e., the first 1000nt of 3′-UTR regions were considered for motif prediction). We found that replacing considered 3′-UTR lengths with the defined lengths by Siegel et al. (8) has no effect on the significance state of 88 predicted motifs, with only one exception (S15 Fig). It is likely that by considering the whole 3′-UTR lengths instead of the truncated version, the approach will predict more motifs that may not be biologically relevant.

RNAcompete

RNAcompete is a single-cycle competition based approach whereby 240,000 different sequences compete to bind to a single RBP (3, 15). The RRE for the RBP is inferred by considering the affinity of every possible 7-mer for binding to the protein and calculating cognate E and Z-scores.

Recently, RNAcompete delineated the binding preference of 205 different genes from 24 diverse eukaryotes (3). This study also revealed that RBPs with similar RNA binding domains (more than 70% identity) typically have similar binding preferences. This observation suggested that binding site information for one RBP could be reliably transformed to other RBPs with a conserved RNA binding domain. However, because of the early-branching of Kinetoplastids in evolution from other eukaryotes, the binding preferences of their RBPs are slightly different from their homologs in other metazoans (3). Therefore, to validate the human results, we examined the similarity of each GRAFFER predicted motif to all RNAcompete motifs,

excluding Kinetoplastids. In the same way, we compared GRAFFER motifs derived from kinetoplastids with the identified RREs of these organisms.

To determine if a GRAFFER motif represents significant similarity with an RNAcompete motif, we set two criteria: 1) sequences containing the computationally predicted motif should be preferentially bound by the corresponding RBP; 2) both RNAcompete and GRAFFER motifs should show similarity at the sequence level, ensuring they both target a similar set of genes. To measure the preference for binding, RNAcompete probes containing the GRAFFER motif were identified and their preferences were examined using the Mann-Whitney sum of ranks test statistic (Benjamini–Hochberg corrected p-value cut-off threshold of 0.05). To consider the similarity with the RNAcompete motifs, we extracted the consensus pattern of each RNAcompete motif, represented in the IUPAC-ambiguity codes. We then determined the enrichment of a predicted motif in an RNAcompete assay as valid, only if the well conserved region, i.e. the discriminative part, of the RNAcompete consensus pattern shared common sequences with the predicted motif. The well-conserved region of a consensus pattern is defined as the region comprising all one and two-degenerate positions (A,U,C,G, S=[CG], W=[AU], Y=[CU], R=[AG], M=[AC], K=[GU]). For example, the conserved region of RNCMPT00138 (from an RNAcompete assay) with consensus pattern of XXVUGAV is XXVUGA. However, the highly degenerate parts of computationally predicted motifs can match with many different conserved regions derived from different RNAcompete assays. For example, computational motifs that contain the fully degenerate sequence of length five (NNNNN), share common sequences with all well conserved regions of length five. To address this issue, we defined a degeneracy rate measure as the entropy of the part of a computational motif that matches with a well-conserved region divided by the entropy of a fully degenerate sequence with the same

length. We only accepted matches with a degeneracy rate of below 50%. In cases where more than one GRAFFER motif matched to the RNAcompete assay, the motif with the highest enrichment was selected.

Comparison with previous studies

To evaluate the performance of our graph-based approach, we compared the GRAFFER results with three other genome-wide studies conducted on *T. brucei* (16-18). It is important to note that RREs are not extensively characterized in *T. brucei*; which forced us to compare the results of each study with a limited set of previously known RREs. Therefore, some of the novel RREs predicted by these approaches may be valid, but not discovered yet. Two of these studies applied the FIRE program (2) in different contexts to predict RREs. FIRE is an information theory-based approach that seeks informative RREs from clusters of co-expressed genes. An independent experimental study showed that the predicted motifs for human are of high quality (19). The third study applied an alignment-free approach, which benefits from simultaneous consideration of four closely related Trypanosomatid species: *T. brucei*, *T. cruzi*, *T. vivax* and *T. congolence*.

In the first genome wide analysis of *T. brucei* genes, the lack of genome-wide experiments available at the time caused the authors to predict “function-specific” RREs by clustering genes according to their function (16). This analysis led to the identification of 21 RREs in the 3′-UTRs of *T. brucei* genes. Considering the same criteria as applied for the GRAFFER motifs, four out of the 21 predicted motifs showed significant similarity with only four different RNAcompete motifs (S4 Table). Predictions did not match with other experimentally-derived motifs.

In the second genome-wide analysis of *T. brucei* genes, whole genome microarray data was available; therefore, the authors employed a sophisticated approach for direct integration of

transcriptome measurements obtained from three independent studies (17). Importantly, two of the transcriptome datasets used in the study are also used for predictions of RREs in our approach. Clustering of the co-expression network and application of FIRE algorithm in this case had led to the prediction of 14 RREs. Comparison with RNAcompete results revealed that three of the 14 predicted motifs showed significant similarity with only three different RNAcompete motifs (S4 Table). Predictions did not match with other experimentally-derived motifs.

In the third genome-wide analysis of *T. brucei* genes, a novel algorithm (COSMOS) was developed on the assumption that orthologous genes in close organisms tend to have a similar set of RREs (18). Application of COSMOS on four closely related Trypanosomatid organisms revealed 222 linear and 166 structural motifs that are conserved among these four organisms. Comparison with RNAcompete results revealed that nine of the 388 predicted motifs had significant similarity with nine different RNAcompete motifs (S4 Table). However, considering the GRAFFER and COSMOS motifs that matched to the same RNAcompete motif, in all cases the GRAFFER motifs showed higher selectivity (higher enrichment) than the COSMOS motifs. It should be pointed that COSMOS was also able to identify three further well-studied motifs. One of them is a structural motif that could not be predicted in the current implementation of GRAFFER algorithm (GRAFFER only searches for linear motifs). The other two are cell cycle-related motifs. GRAFFER successfully discovered one of these motifs from the transcriptome data of cell cycle progression (see above). However, the second motif is related to a set of transcripts with subtle variations in their expression, as mentioned in (6). In our motif prediction pipeline, we constructed co-expression graphs by focusing on highly variable genes. Therefore, we most probably missed this motif because we did not have its cognate targets in the co-expression graph.

Supplementary Figures

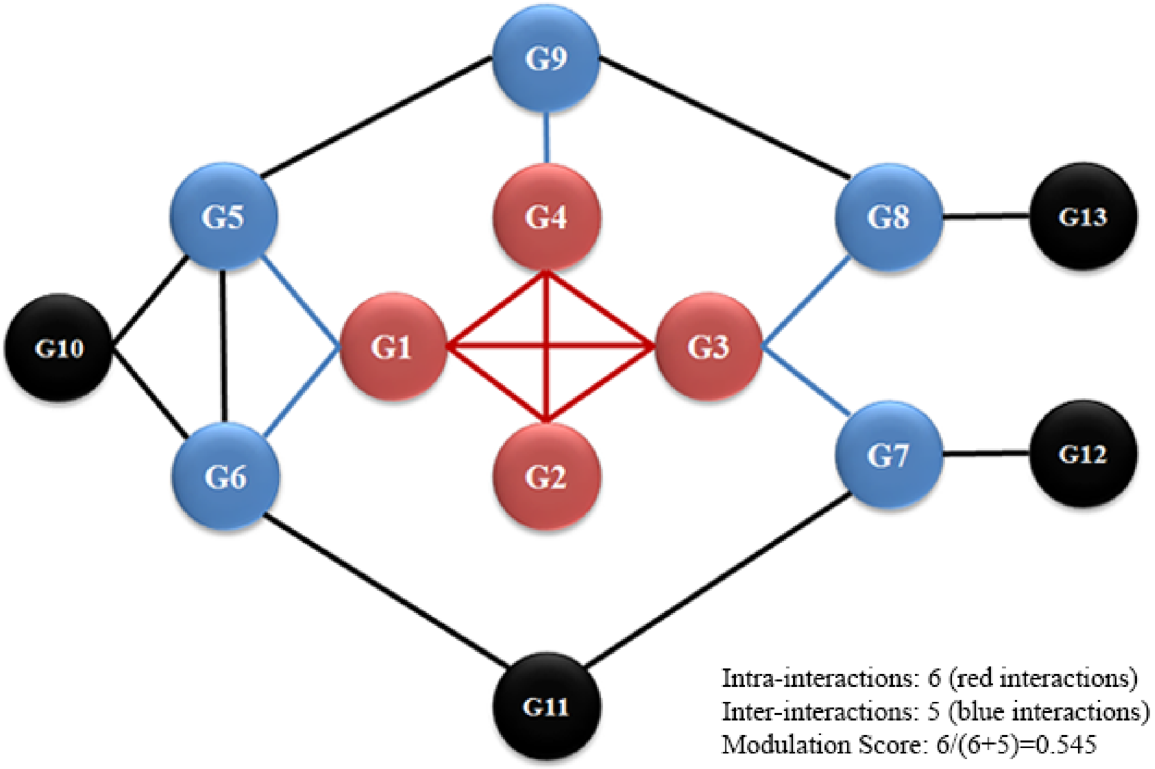

S1 Fig. Schematic representation of the defined motif modulation score.

Red vertices represent transcripts containing at least one instance of the motif in their 3'-UTR, blue vertices represent first neighbors of red nodes in the co-expression graph. Black vertices represent the other genes in the graph. Intra-interactions (red edges) are defined as interactions among red vertices. Inter-interactions (blue edges) are defined as interactions between red vertices and blue vertices in the co-expression graph. The modulation score measures the connectivity density of targeted genes by a motif. For the illustrative purposes, all edge weights are considered equal to one.

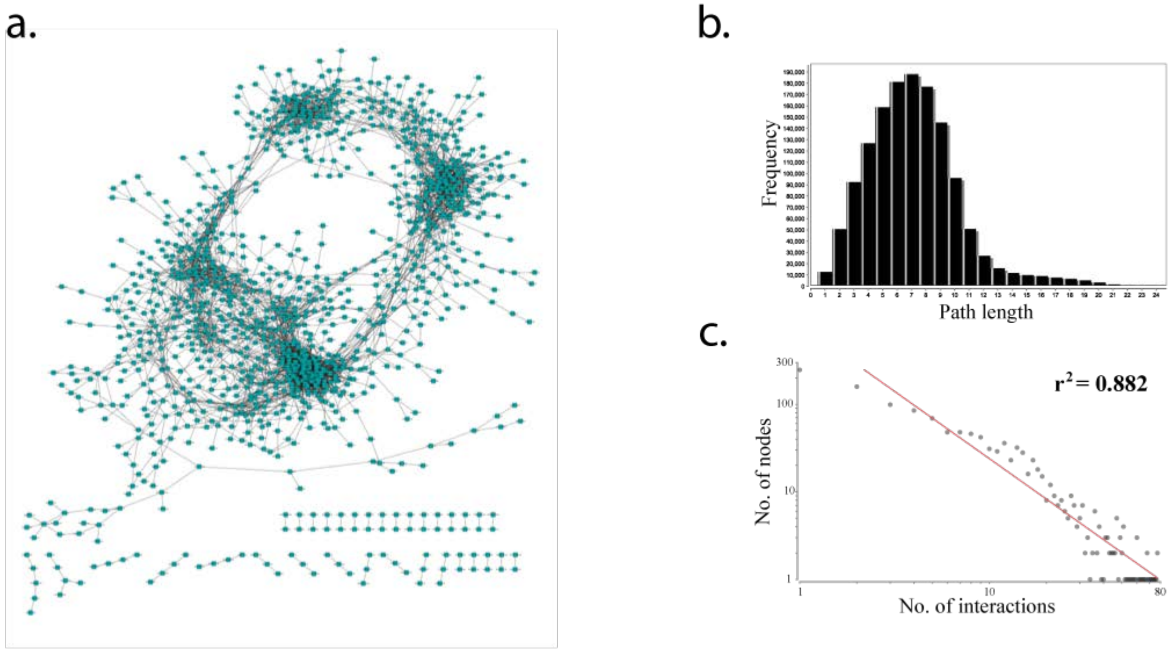

S2 Fig. Constructed Co-expression graph based on three independent transcriptome datasets.

(**a**) Global view of the dichotomized co-expression graph for *T. brucei* genes, based on the integration of transcriptome data from three independent studies. The constructed graph is modular, i.e. there are highly connected regions in the graph that are separated from the other parts. The constructed co-expression graph has **(b)** scale-free and **(c)** small-world architecture.

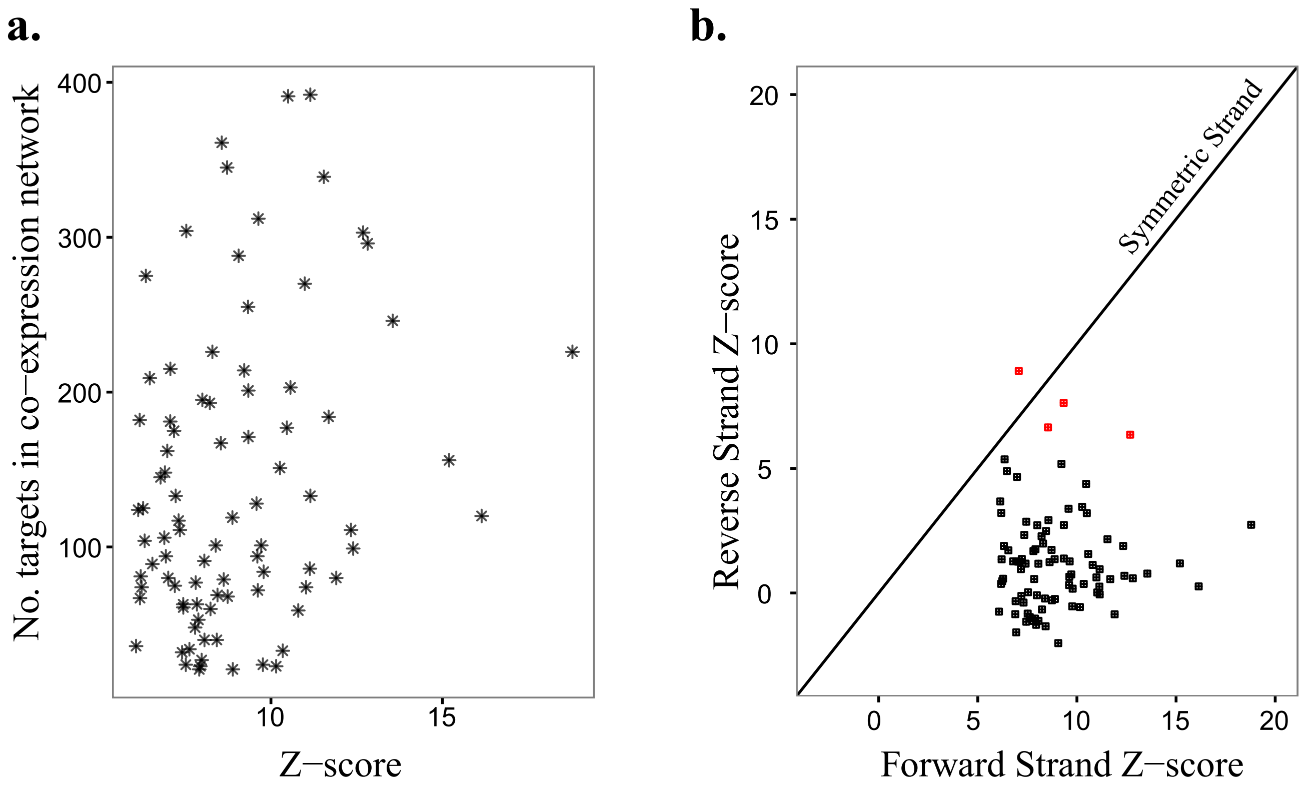

S3 Fig. Characteristics of motifs which were predicted from the integrated co-expression graph of *T. brucei.*

(a) Z-scores and the number of targets for significant motifs in the *T. brucei* integrated co-expression graph. **(b)** Strand bias analysis of GRAFFER motifs. Eighty-four motifs (black nodes) were only significant in the forward strand; while, only four motifs (red nodes) were significant in both strands.

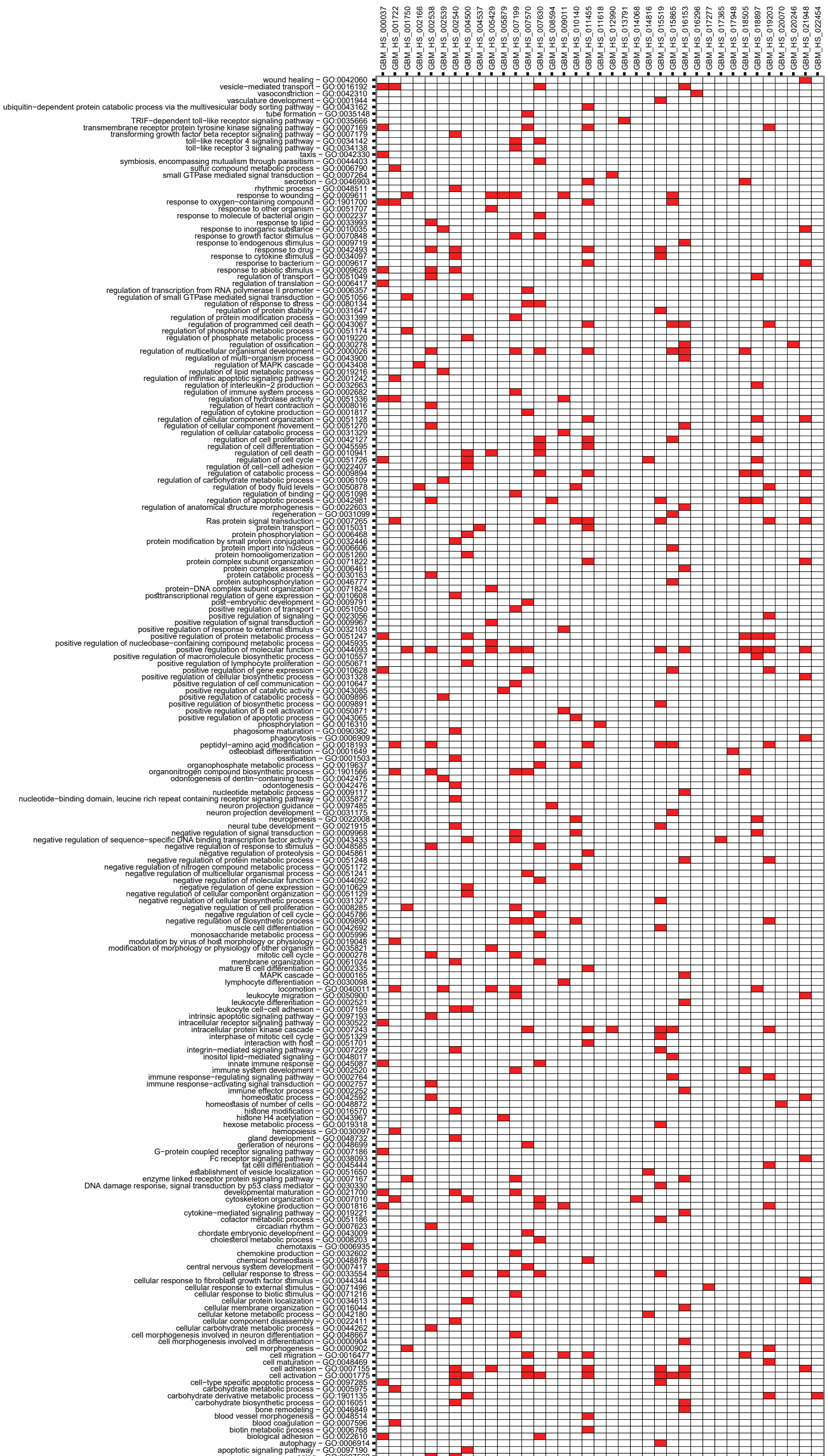

S4 Fig. Gene ontology biological process (GO-BP) enrichment analysis for predicted motifs based on human co-expression graph.

We used g:profiler web server for enrichment analysis (24). In our analysis, we only considered categories with between 50 upto 1500 annotated genes. Each module was analyzed independently and enriched terms with Benjamini corrected p-value less than 0.01 were selected. To avoid redundant GO-BP terms, “Best per parent group” filtering option was chosen.

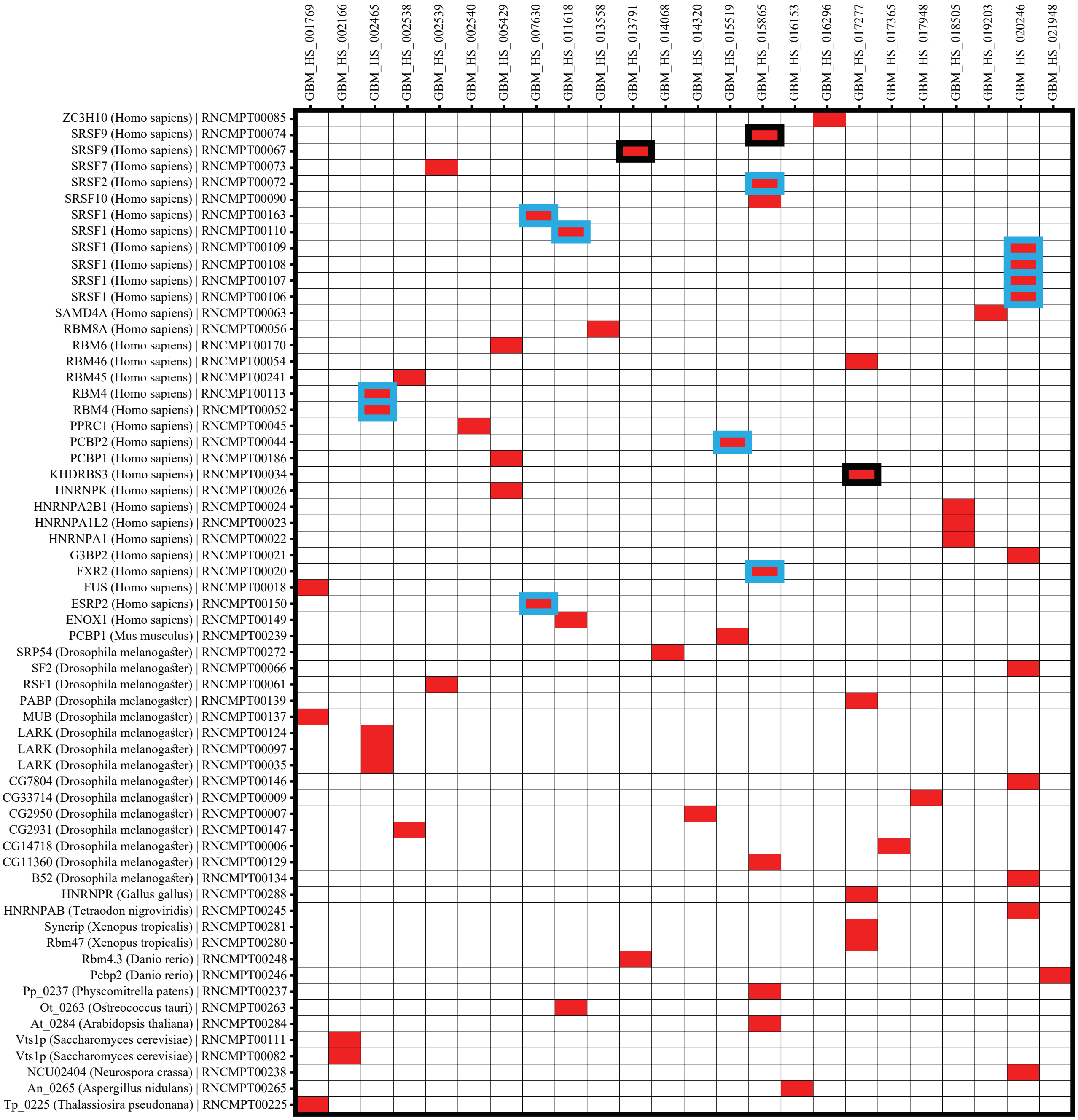

S5 Fig . Comparison of predicted motifs for human with the identified RREs in recent large scale RNAcompete experiment.

24 out of 49 predicted motifs show significant similarty with identified RREs in 62 RNAcompete experiments (some of the RNAcompete motifs are highly similar to each other because of the existence of experimental replicates and/or conserved RNA binding domains). The bold blue frame indicates cases in which the GRAFFER motif was enriched (two tailed hypergeometric, p-value <0.01) among the RNA targets of the RBP as reported in (3); and the bold black frame indicates cases where GRAFFER motif was not enriched among the target RNAs.

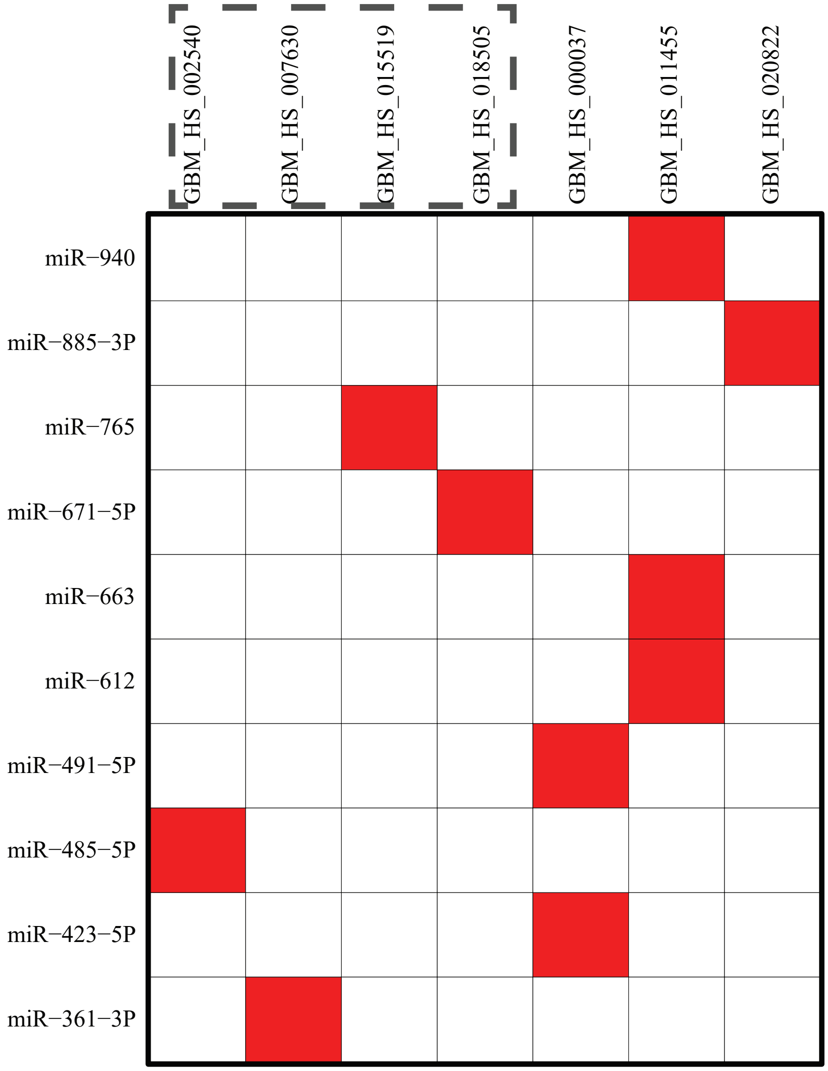

S6 Fig. Comparison of GRAFFER motifs with the known human miRNAs

To examine whether or not the predicted motifs by GRAFFER can be the binding site of human miRNAs, we set two criteria: 1) The genes that harbor the motif should be enriched for the potential targets of a human miRNA. We used g:profiler web server for this analysis (24); 2) The 5′-extermity of the miRNA should match to the reverse complement of the predicted motif sequence, allowing at most two nucleotide shifts in either miRNA or motif sequence. As illustrated, we found 7 motifs potentially represent the binding sites for 10 human miRNAs. Note that some of the predicted motifs not only can match with miRNAs, but also they can represent the binding site of RBPs (highlighted with the box).

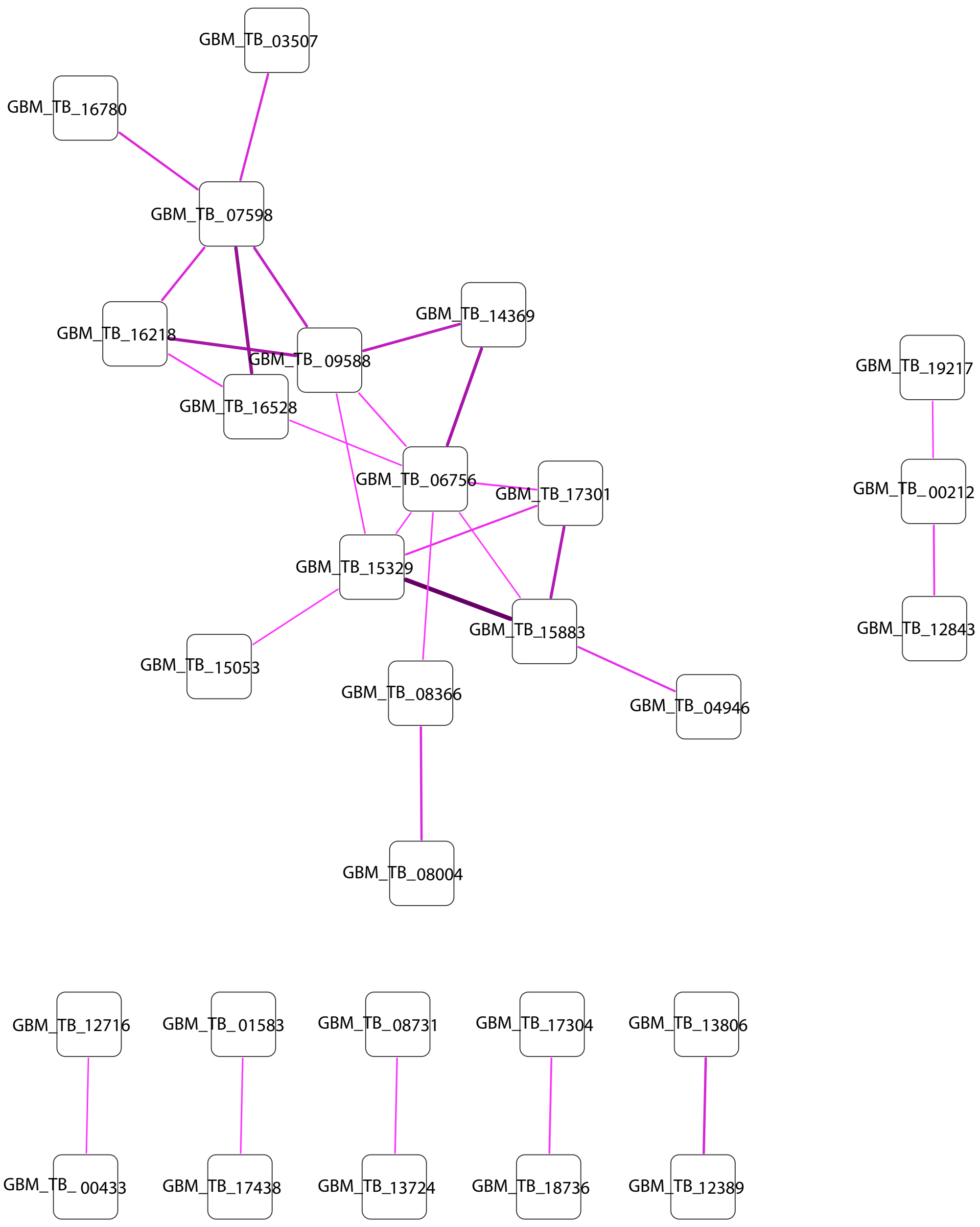

S7 Fig. The motif co-occurrence network for 88 predicted motifs based on *T. brucei* co-expression graph

Motif co-occurrence profile represented as a network. Different RBPs can regulate the same set of transcripts. These combinatorial regulatory networks were captured by determining if the targeted genes by two different motifs significantly overlap with each other. The color density represents the calculated Z-scores for each interaction.

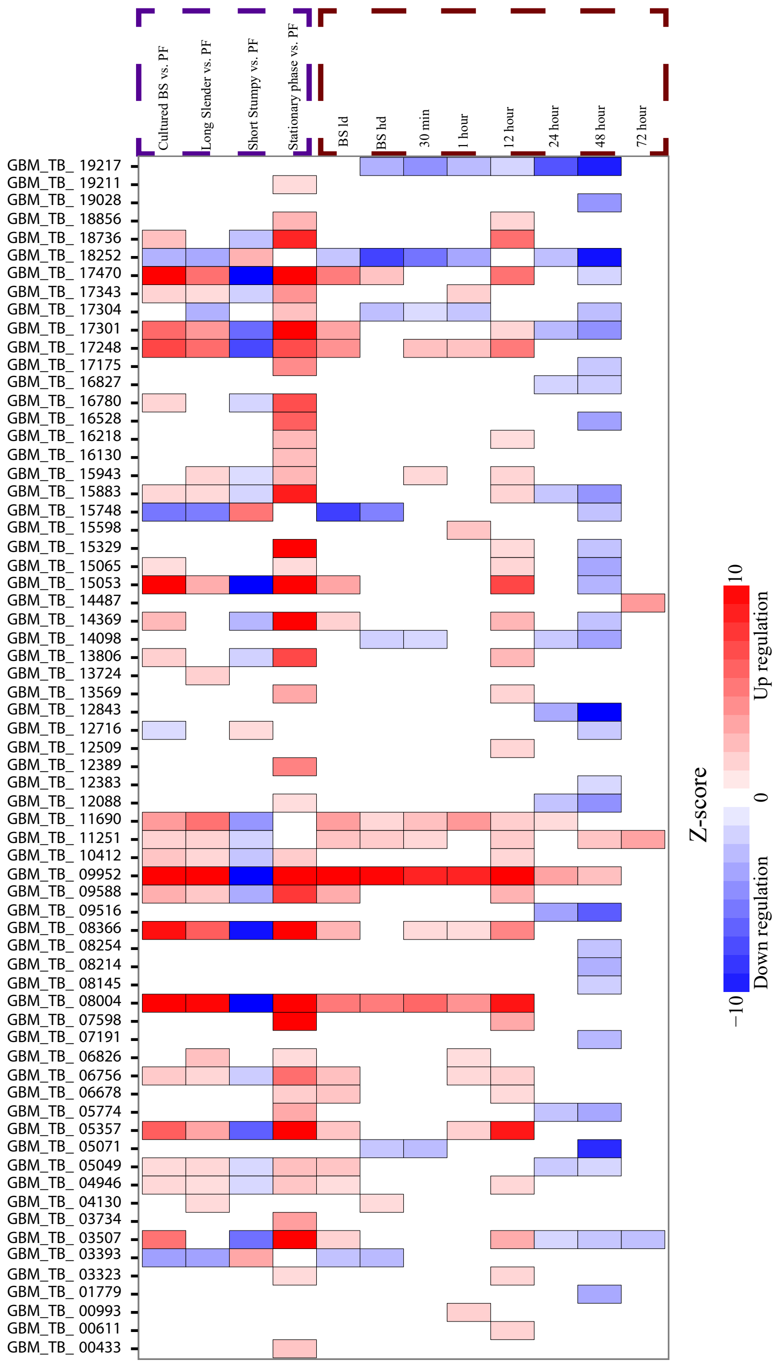

S8 Fig. Enrichment analysis of GRAFFER motifs in different cell states.

Predicted motifs are responsive to the developmental transcriptome changes. Motif names are shown on the left and biological conditions on the top. The motif enrichment scores are represented in pseudo-colors, with only significant scores shown (Mann-Whitney rank sum, 5% FDR threshold). Biological conditions in the red box are related to the differentiation process from the bloodstream form to the procyclic form. Biological conditions in the purple box are related to the different life stages of *T. brucei*. BS hd = bloodstream high density, representing the short stumpy form. BS ld = bloodstream low density, representing the long slender form.

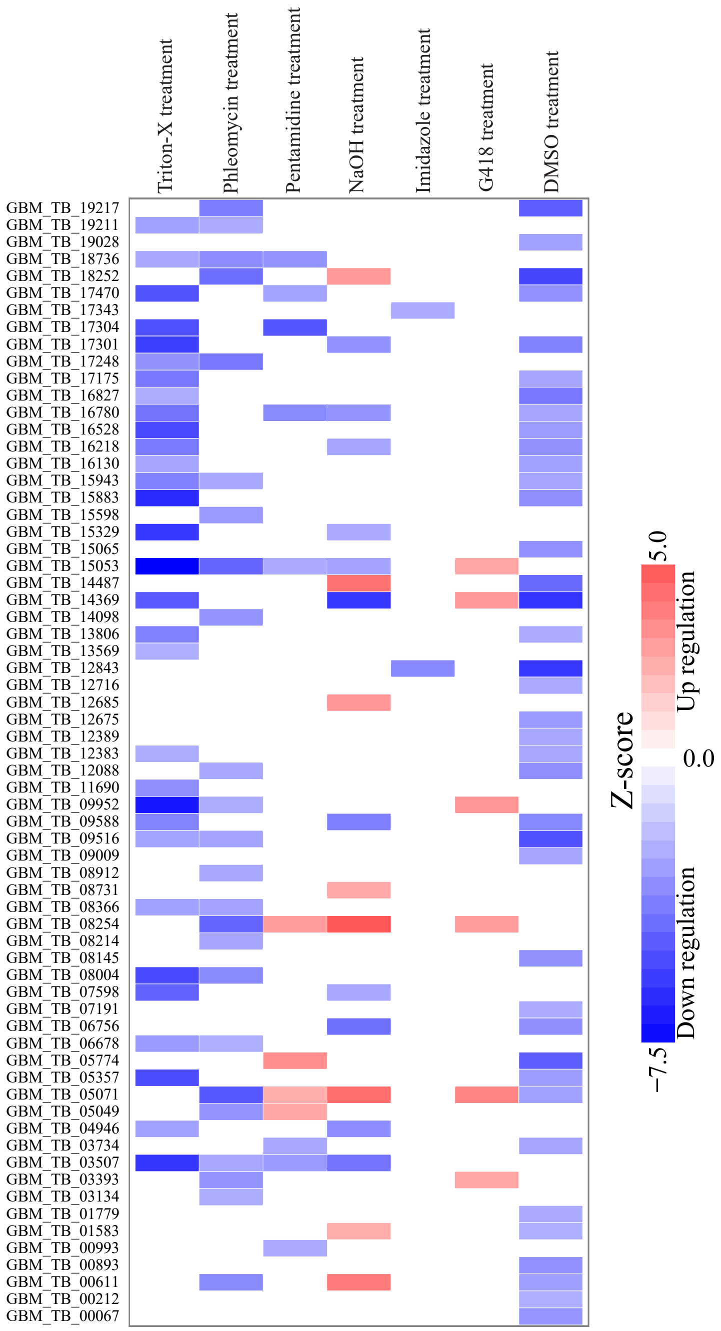

S9 Fig. Enrichment analysis of GRAFFER motifs in response to different chemical stresses.

The figure is pseudo-colored, only significant enrichments are presented (Mann-Whitney rank sum, 5% FDR threshold). We have excluded four stress conditions (EtBr treatment, HCL treatment, Hygromycin treatment, and Verapamil treatment) from the enrichment analysis because the selected highly variable genes were coherently up- or downregulated under these conditions. Therefore, the enrichment analysis of motifs that were predicted based on these genes would show a bias.

a.

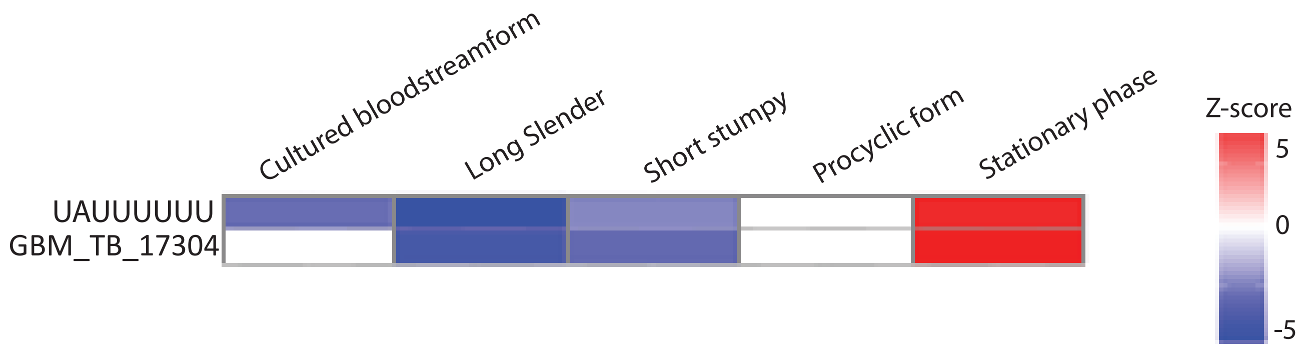

b.

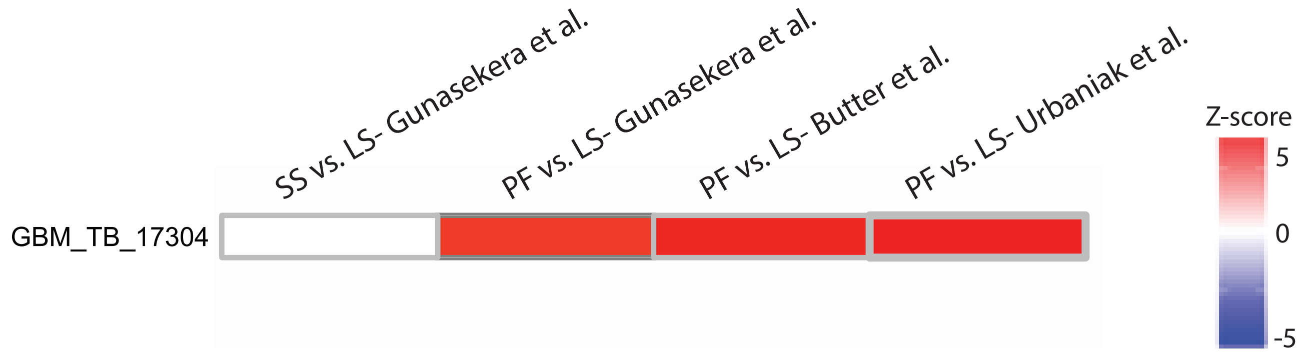

S10 Fig Developmentally regulated RRE in *T. brucei.*

Comparison of an experimentally established RRE (UAUUUUUU) that is involved in developmental regulation of *T. brucei* genes, with GRAFFER motif, GBM_TB_17304. As shown, both motifs show similar developmental responses. **a)** Transcripts targeted by the experimentally-derived motif or GBM_TB_17304 were selected and then tested for a statistically significant pattern in each cell state using Mann-Whitney rank sum statistic. Expression data were extracted from (20). b) Proteome responses of both motifs to the developmental changes in *T. brucei* were analyzed using Mann-Whitney rank sum statistic. Protein expression data were extracted from Gunasekera et al. (21), Butter et al. (22), and Urbaniak et al. (23).

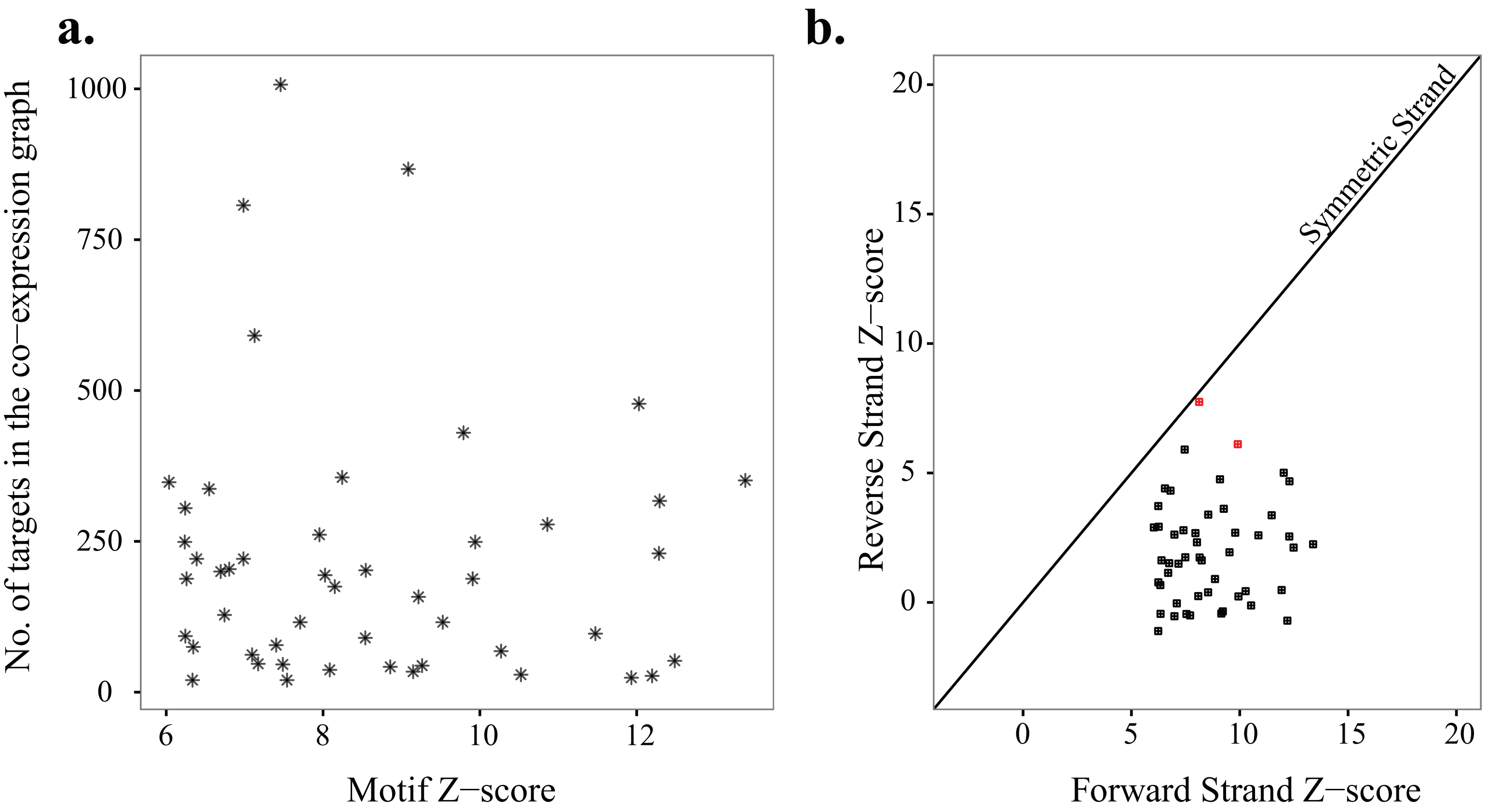

S11 Fig. Characteristics of predicted motifs based on human's co-expression graph.

(a) The distribution of z-scores and the number of targeted genes for each of 49 predicted motifs. (b)The predicted motifs show directionality bias, i.e. they are mostly significant in the forward strand. Black nodes represent those motifs that are significant only in the forward strand. Red nodes indicate the two palindromic motifs that are significant in both forward and reverse strands.

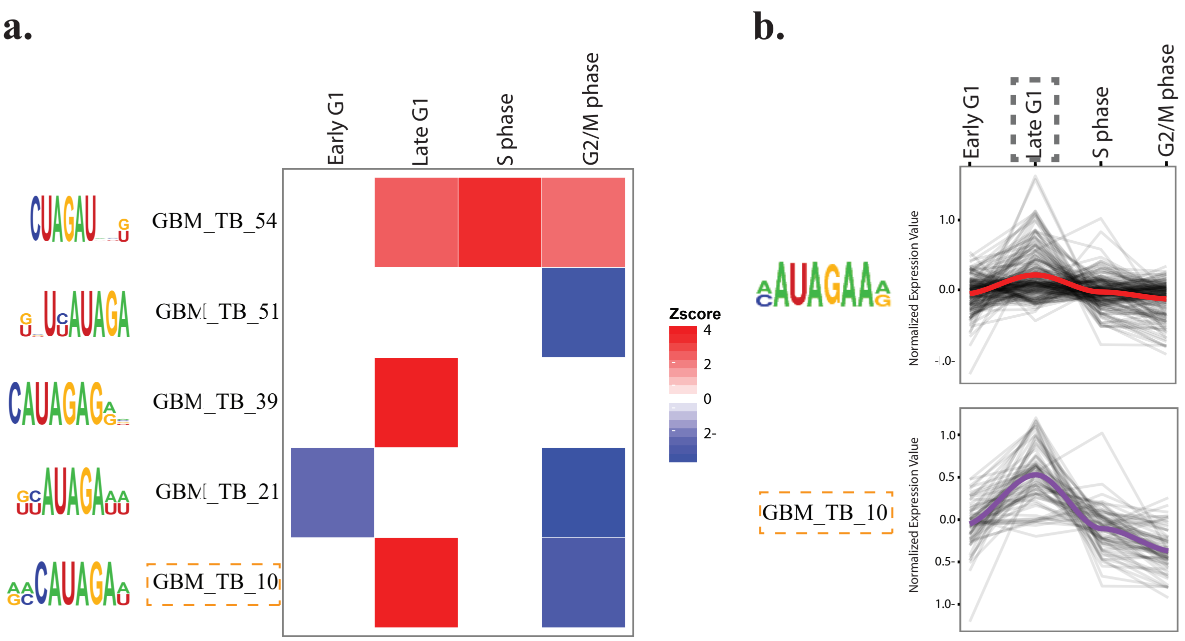

S12 Fig. Transcriptome responses of GRAFFER motifs that were predicted based on the cell cycle transcriptome data of *T. brucei*.

(**a**) Predicted motifs are responsive to the transcriptome changes during cell cycle progression of *T. brucei*. **(b)** Comparison of an experimentally validated RRE, with a role in the cell cycle regulation, with GBM_TB_10. Both motifs are significantly upregulated in late G1 phase (Mann-Whitney rank sum statistic, p-value <0.05). The experimentally established RRE was extracted from (7).

a.

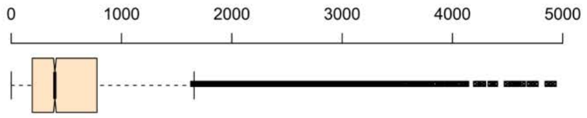

b.

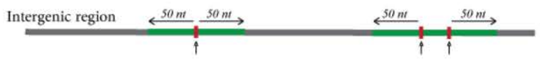

c.

	Kolev et al.	Siegel et al.	Intersection
# PAS	52078	16863	-
# genes ≥ 2regions	2811	1953	1204

d.

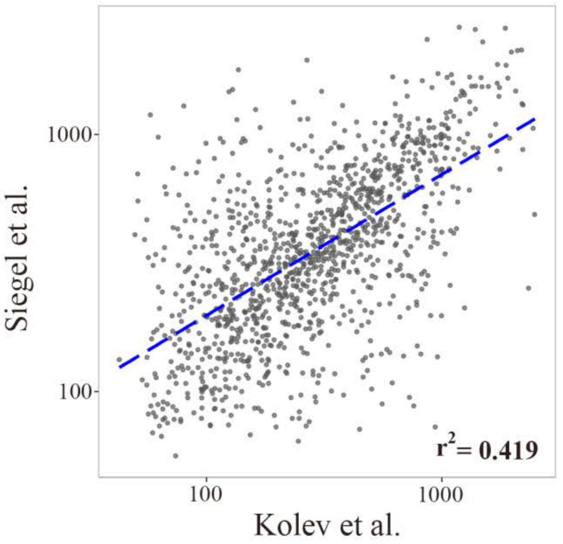

e.

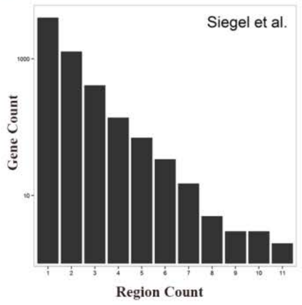

f.

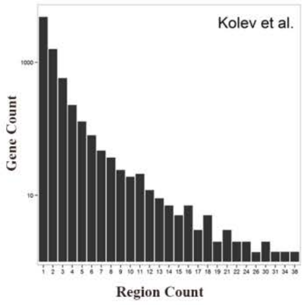

S13 Fig. Characteristics of *T. brucei* 3′-UTRs

(**a**) 3′-UTR length variation of *T. brucei* genes according to Siegel et al (8). In cases where a gene has alternative poly-adenylation sites, the 3′-UTR length is defined as the median length; **(b)** schematic representation of the defined poly-adenylation sites. Upward arrows represent the location of detected poly-adenylation sites for a gene. Each region is defined as 50nt before and after the detected poly-adenylation site. If the distance between two poly-adenylation sites was less than 100nt, the two corresponding regions were merged together. **(c)** Number of poly-adenylation sites and regions determined in two independent studies. As shown, many genes have more than one determined poly-adenylation region. **(d)** Correlation of two studies for 3′-UTR length variation of genes with more than one poly-adenylation region. The Y-axis and X-axis indicate the standard division of 3′-UTR lengths according to Siegel et al. (8) and Kolev et al. (14), respectively. **(e)** Distribution of genes based on the number of poly-adenylation regions, according to Siegel et al. (8) **(f)** Distribution of genes based on the number of poly-adenylation regions, according to Kolev et al. (14).

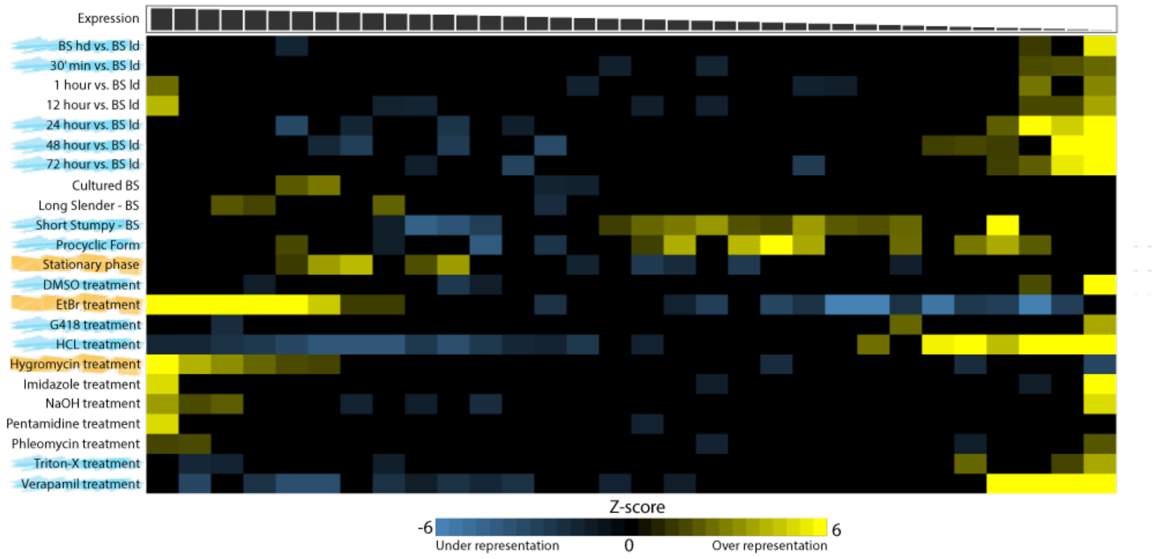

S14 Fig. Patterns of up- and downregulation of genes with long 3′-UTRs under different experimental conditions.

For each condition, genes wee sorted according to their expression value. Sorted genes were then divided into 30 different bins. The enrichment of genes with long 3′-UTRs in each bin was examined using Fisher’s exact test. Yellow color shows over-representation of genes in the corresponding bin. Similarly, blue represents under-representation of these genes in the cognate bin. The figure is pseudo-colored, only statistically significant bins are colored (Bonferroni corrected p-value < 0.05). Highlighted conditions on the left show overall significant up- or downregulation using Mann-Whitney rank sum statistics. Blue backgrounds indicate downregulation and orange backgrounds represent upregulation of genes with long 3′-UTRs.

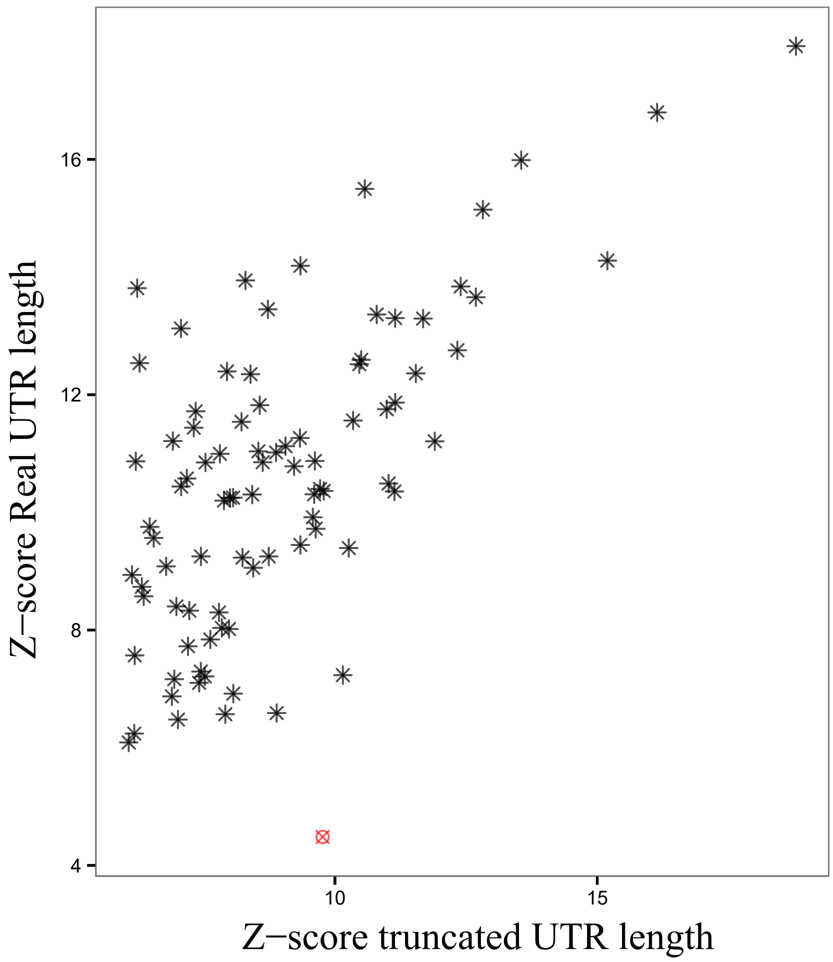

S15 Fig. Significant state of motifs after consideration of full length 3′-UTRs other than the trimmed version.

Except in one case (red node), considering the reported 3′-UTR lengths by Siegel et al. (8) instead of the trimmed versions did not have a noticeable effect on the significance state of most GRAFFER motifs.

References

1. Novershtern N, Subramanian A, Lawton LN, Mak RH, Haining WN, McConkey ME, et al. Densely interconnected transcriptional circuits control cell states in human hematopoiesis. Cell. 2011;144(2):296-309.
2. Elemento O, Slonim N, Tavazoie S. A universal framework for regulatory element discovery across all genomes and data types. Mol Cell. 2007;28(2):337-50.
3. Ray D, Kazan H, Cook KB, Weirauch MT, Najafabadi HS, Li X, et al. A compendium of RNA-binding motifs for decoding gene regulation. Nature. 2013;499(7457):172-7.
4. van Kouwenhove M, Kedde M, Agami R. MicroRNA regulation by RNA-binding proteins and its implications for cancer. Nature reviews Cancer. 2011;11(9):644-56.
5. Ho JJ, Marsden PA. Competition and collaboration between RNA-binding proteins and microRNAs. Wiley Interdiscip Rev RNA. 2014;5(1):69-86.
6. Archer SK, Inchaustegui D, Queiroz R, Clayton C. The cell cycle regulated transcriptome of Trypanosoma brucei. PloS one. 2011;6(3):e18425.
7. Bhandari D, Guha K, Bhaduri N, Saha P. Ubiquitination of mRNA cycling sequence binding protein from Leishmania donovani (LdCSBP) modulates the RNA endonuclease activity of its Smr domain. FEBS letters. 2011;585(5):809-13.
8. Siegel TN, Hekstra DR, Wang X, Dewell S, Cross GA. Genome-wide analysis of mRNA abundance in two life-cycle stages of Trypanosoma brucei and identification of splicing and polyadenylation sites. Nucleic acids research. 2010;38(15):4946-57.
9. Elkon R, Drost J, van Haaften G, Jenal M, Schrier M, Vrielink JA, et al. E2F mediates enhanced alternative polyadenylation in proliferation. Genome biology. 2012;13(7):R59.
10. Mayr C, Bartel DP. Widespread shortening of 3'UTRs by alternative cleavage and polyadenylation activates oncogenes in cancer cells. Cell. 2009;138(4):673-84.
11. Jager AV, De Gaudenzi JG, Cassola A, D'Orso I, Frasch AC. mRNA maturation by two-step trans-splicing/polyadenylation processing in trypanosomes. Proceedings of the National Academy of Sciences of the United States of America. 2007;104(7):2035-42.
12. Nilsson D, Gunasekera K, Mani J, Osteras M, Farinelli L, Baerlocher L, et al. Spliced leader trapping reveals widespread alternative splicing patterns in the highly dynamic transcriptome of Trypanosoma brucei. PLoS pathogens. 2010;6(8):e1001037.
13. Matthews KR, Tschudi C, Ullu E. A common pyrimidine-rich motif governs trans-splicing and polyadenylation of tubulin polycistronic pre-mRNA in trypanosomes. Genes & development. 1994;8(4):491-501.
14. Kolev NG, Franklin JB, Carmi S, Shi H, Michaeli S, Tschudi C. The transcriptome of the human pathogen Trypanosoma brucei at single-nucleotide resolution. PLoS pathogens. 2010;6(9):e1001090.
15. Ray D, Kazan H, Chan ET, Pena Castillo L, Chaudhry S, Talukder S, et al. Rapid and systematic analysis of the RNA recognition specificities of RNA-binding proteins. Nature biotechnology. 2009;27(7):667-70.
16. Mao Y, Najafabadi HS, Salavati R. Genome-wide computational identification of functional RNA elements in Trypanosoma brucei. BMC genomics. 2009;10:355.
17. Shateri Najafabadi H, Salavati R. Functional genome annotation by combined analysis across microarray studies of Trypanosoma brucei. PLoS neglected tropical diseases. 2010;4(8).
18. Najafabadi HS, Lu Z, MacPherson C, Mehta V, Adoue V, Pastinen T, et al. Global identification of conserved post-transcriptional regulatory programs in trypanosomatids. Nucleic Acids Res. 2013;41(18):8591-600.

19. Hu S, Xie Z, Onishi A, Yu X, Jiang L, Lin J, et al. Profiling the human protein-DNA interactome reveals ERK2 as a transcriptional repressor of interferon signaling. Cell. 2009;139(3):610-22.
20. Jensen BC, Sivam D, Kifer CT, Myler PJ, Parsons M. Widespread variation in transcript abundance within and across developmental stages of Trypanosoma brucei. BMC genomics. 2009;10:482.
21. Gunasekera K, Wuthrich D, Braga-Lagache S, Heller M, Ochsenreiter T. Proteome remodelling during development from blood to insect-form Trypanosoma brucei quantified by SILAC and mass spectrometry. BMC genomics. 2012;13:556.
22. Butter F, Bucerius F, Michel M, Cicova Z, Mann M, Janzen CJ. Comparative proteomics of two life cycle stages of stable isotope-labeled Trypanosoma brucei reveals novel components of the parasite's host adaptation machinery. Molecular & cellular proteomics : MCP. 2013;12(1):172-9.
23. Urbaniak MD, Guther ML, Ferguson MA. Comparative SILAC proteomic analysis of Trypanosoma brucei bloodstream and procyclic lifecycle stages. PloS one. 2012;7(5):e36619.
24. Reimand J, Arak T, Vilo J. g:Profiler--a web server for functional interpretation of gene lists (2011 update). Nucleic Acids Res. 2011;39(Web Server issue):W307-15.
